# Diversity of *Cytospora* Species Associated with Trunk Diseases of *Prunus persica* (Peach) in Northern China

**DOI:** 10.3390/jof10120843

**Published:** 2024-12-05

**Authors:** Zhizheng He, Pranami D. Abeywickrama, Linna Wu, Yueyan Zhou, Wei Zhang, Jiye Yan, Qiaoxia Shang, Ying Zhou, Shifang Li

**Affiliations:** 1 Beijing Key Laboratory of Environment Friendly Management on Fruit Diseases and Pests in North China, Key Laboratory of Environment Friendly Management on Fruit and Vegetable Pests in North China (Co-Construction by Ministry and Province), Ministry of Agriculture and Rural Affairs, Institute of Plant Protection, Beijing Academy of Agriculture and Forestry Sciences, Beijing 100097, China; 1873039722z@gmail.com (Z.H.); pranamiabeywickrama@yahoo.com (P.D.A.); guiwawa4567@126.com (L.W.); yueyan_zhou@163.com (Y.Z.); zhwei1125@163.com (W.Z.); jiyeyan@vip.163.com (J.Y.); 2 Key Laboratory for Northern Urban Agriculture of Ministry of Agriculture and Rural Affairs, College of Bioscience and Resource Environment, Beijing University of Agriculture, Beijing 102206, China; 3 Center of Excellence in Fungal Research, Mae Fah Luang University, Chiang Rai 57100, Thailand; 4 School of Science, Mae Fah Luang University, Chiang Rai 57100, Thailand; 5 State Key Laboratory for Biology of Plant Diseases and Insect Pests, Institute of Plant Protection, Chinese Academy of Agricultural Sciences, Beijing 100193, China; sfli@ippcaas.cn

**Keywords:** fungi, gummosis, morphology, pathogenicity, peach Cytospora canker, phylogeny

## Abstract

Peach (*Prunus persica*) is widely cultivated in China, but fungal diseases, particularly Cytospora canker, significantly impact tree health, reducing fruit yield and economic value. This disease mainly weakens tree branches and trunks, sometimes leading to tree death. There are no updated studies on the diversity of *Cytospora* species associated with peach Cytospora canker in northern China. To determine the *Cytospora* species associated with this disease, we surveyed five provinces from 2022 to 2023, collecting 72 disease samples with symptoms including branches with black fruiting bodies, cankers, cracking, dieback, and gummosis. Through morphology and multi-loci phylogeny, 127 isolates were identified into four known (*C. ailanthicola*, *C. erumpens*, *C. leucostoma*, and *C. leucosperma*) and two previously undescribed species (*C. gansuensis* sp. nov. and *C. qinanensis* sp. nov.). *Cytospora leucostoma* (73.60%) was the most abundant. Pathogenicity tests indicated that except for *C. ailanthicola*, all other species were pathogenic to peach, with *C*. *erumpens* being the most aggressive. This study is the first to report the novel host association of *C. erumpens* on peaches globally and represents the first comprehensive investigation of *Cytospora* species associated with canker diseases in the main peach production area in northern China, offering a foundation for developing effective disease management strategies.

## 1. Introduction

Peaches (*Prunus persica* L.) are a fruit that belongs to the family Rosaceae, and they are generally liked because of their exotic taste, colour and positive implications on health [1,2]. China is the native home for peach, mainly in plateau regions of Shaanxi, Gansu, and other parts of China, at altitudes of 900–3150 m [3], and it has more than 3000 years of cultivation history [4,5]. This fruit crop has both economic and esthetic value as an ornamental plant and it is considered one of the most commonly grown stone fruits in China [2,3,6].

Peaches are widely cultivated across China, with primary cultivation areas in the north, northwest, and southwest regions [7]. According to the data from the Food and Agricultural Organization, China’s peach planting area and yield have steadily increased from 2013 to 2022, making China the world’s largest peach producer (FAO 2024). Even though the relatively low temperatures and snow have damaged peach flower blossoms in northern China in previous years, southern Chinese provinces have shown favourable growing conditions. Therefore, China’s peach production was estimated at 17.5 million metric tons (MMT) in the last marketing year (2023/2024) (USDA—Stone Fruit Annual 2023). Most peach-growing areas in China still rely on traditional methods, and the management is still relatively extensive, which leads to frequent disease outbreaks and limits the high-quality production of peaches [8,9]. Fungal diseases, especially cankers, die-backs, and gummosis, are major issues and are considered the main biotic factor that limits the fruit production of peaches in China [2].

Cytospora canker is a common trunk and branch disease in stone fruits, including peaches. It mainly affects trunks and branches [10,11] and can severely weaken or kill trees, reducing yield and economic value [12]. Peach Cytospora canker widely occurs around the world. In China, this disease is primarily observed in northern regions like Liaoning, Hebei, Shandong, and Shaanxi [13], where cold winters create favourable conditions for infection [14]. This disease is mainly caused by *Cytospora* species, such as *C. leucostoma* and *C. leucosperma* [11,15,16,17,18,19]. The first international report of peach Cytospora canker was reported in 1925 in the United States [20] and the first Chinese record was in 1960 [21]. The USDA Fungus-Host Database lists 27 *Cytospora* species associated with peach trees worldwide, but only two (*Cytospora japonica* and *Cytospora leucostoma*) are recorded in China (USDA Fungus-Host Database https://fungi.ars.usda.gov/, accessed on 5 July 2024).

*Cytospora* belongs to the Cytosporaceae family in Sordariomycetes (MycoBank, 2024) [22] and this genus was established by Ehrenberg [23]. *Cytospora* species are widely distributed around the world and are known as one of the most important phytopathogenic genera causing trunk diseases on various woody plant hosts [24,25,26,27,28,29]. Currently, there are 769 *Cytospora* records listed in Index Fungorum (Index Fungorum 2024 https://www.indexfungorum.org/names/names.asp, accessed on 25 November 2024) and among them, about 500 are reported as plant pathogens [30]. *Cytospora* species infect over 85 plant hosts, especially Salicaceae and Rosaceae [29,31,32,33]. Previously, *Cytospora* species were identified based on host associations and morphological features. However, the morphology of many *Cytospora* species does not provide significant differences; thus, relying solely on morphology-based identification is more dubious. Currently, the identification and classification of *Cytospora* species mainly rely on multi-gene phylogenetic analysis and morphological observations [34]. By 2020, 88 *Cytospora* species had been recorded in China [35], but many are vaguely described [36].

Despite the significant impact of peach Cytospora canker in China [13], research on peach trunk diseases and their causative organisms has been poorly studied. Therefore, to identify and characterize the *Cytospora* species associated with peach Cytospora canker disease, we surveyed five peach-growing regions in northern China. Using morpho-molecular approaches, we characterized the fungal species and determined the pathogenicity of a subset of these identified species.

## 2. Materials and Methods

### 2.1. Disease Symptoms, Sample Collection and Fungal Isolation

**Figure 1:**
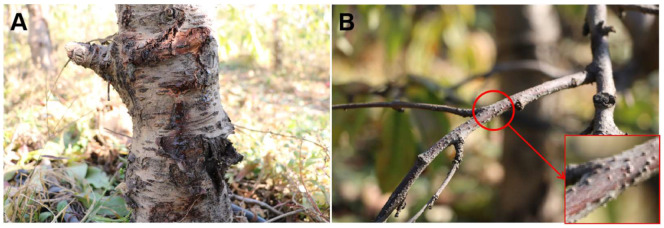
Field symptoms of peach Cytospora canker. (**A**) The peach trunk shows cracks and exudating gum (Gummosis). (**B**) The *Cytospora* fruiting bodies on the peach branches.

**Figure 2:**
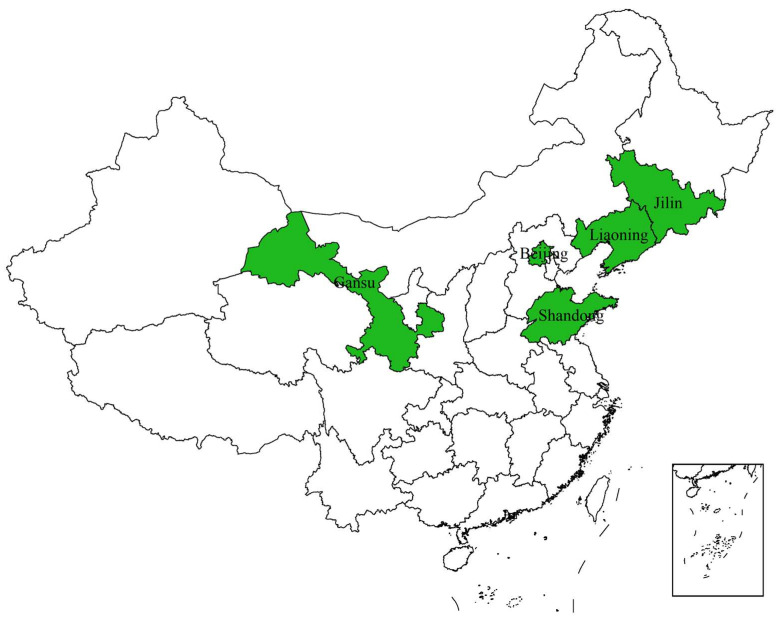
Collection sites of peach Cytospora canker samples in China. The collection sites are marked in green.

From June 2022 to July 2023, peach branch, trunk and twig samples exhibiting Cytospora canker symptoms (including canker, gummosis, twig blight, diebacks, cracks, and branches with black fruiting bodies) were collected (Figure 1) from five regions in northern China (Beijing, Gansu, Liaoning, Jilin, and Shandong) (Figure 2). Symptomatic samples were carefully removed from trees and placed in Zip-lock bags and their field symptoms, collection date and locations were recorded. The samples were transported to the laboratory and stored at 4 °C until further examination.

Fungal isolation was performed using a modified version of the method described by Senanayake et al. [37]: (i) Diseased samples without any fungal fruiting structures employed a conventional tissue separation method [37]. A small piece between healthy and diseased tissues was cut and washed with 75% alcohol for 30 s for surface sterilization. Then, tissue pieces were washed three times with sterile distilled water and placed on Petri plates containing potato dextrose agar (PDA). Plates were incubated at 25 °C for three days and hyphae growing from the tissue edges were transferred to new PDA plates. Pure cultures were obtained through single hyphal or spore isolation. (ii) Diseased samples exhibiting fungal fruiting structures were handled as follows: branches with fruiting bodies were surface-sterilized, and fruiting bodies were picked and transferred to a microcentrifuge tube containing sterile distilled water to prepare the spore suspension. After observation under a microscope and confirming the availability of spores, 100 μL of spore suspension was spread over the surface of water agar (WA) plates. After 12–24 h of incubation at 25 °C, single germinating spores were transferred to new PDA plates.

All pure cultures obtained in this study were deposited in the culture collection of the Institute of Plant Protection, Beijing Academy of Agriculture and Forestry Sciences (JZB), China. Herbarium materials including holotypes of novel species were deposited as dry cultures in the herbarium of the Institute of Plant Protection, Beijing Academy of Agriculture and Forestry Sciences (JZBH), China.

### 2.2. DNA Extraction, PCR Amplification and Sequencing

Fungal isolates grown on PDA plates for 7 days at 25 °C were used to collect the mycelia for DNA extraction. Total genomic DNA was extracted from purified fungal colonies using a TaKaRa MiniBEST Plant Genomic DNA Extraction Kit (Takara, Japan) according to the manufacturer’s protocol. Five gene regions (ITS, LSU, *rpb2*, *tef1-α*, and *tub2*) were amplified by using respective primers. The primer pairs and protocols used in this study are summarized in Table 1. The PCR reactions were carried out in 25 μL volumes containing 12.5 μL of 2 × Taq PCR MasterMix (Beijing Biomed Gene Technology Co., Ltd., Beijing, China), 10.5 μL of ddH_2_O, 0.5 μL of forward primer, 0.5 μL of reverse primer, and 1 μL of DNA template. The PCR products were assessed using agarose gel electrophoresis and gel products stained with ethidium bromide were observed under UV light using GelDocXR+ (Bio-Rad Laboratories Inc., Hercules, CA, USA). All the successful PCR products were sequenced at Beijing Qingke Biotechnology Co., Ltd. (Beijing, China).

### 2.3. Phylogenetic Analyses

The electropherogram of newly generated sequences was checked with BioEdit 7.0.9.0 to confirm sequence quality. Reference sequences were downloaded from GenBank (https://www.ncbi.nlm.nih.gov/genbank/, accessed on 26 February 2024), following the relevant literature (Supplementary Table S1). The DNA sequence datasets were aligned using MAFFT v7 (https://mafft.cbrc.jp/alignment/server/, accessed on 28 June 2024) [44] and alignments were manually trimmed using BioEdit v7.0.9.1 where necessary.

Combined sequence data of ITS, LSU, *rpb2*, *tef1-α* and *tub2* gene regions were used for the maximum likelihood (ML) and Bayesian inference (BI) analyses. The maximum likelihood analysis was performed with the RAPxML-HPC2 tool (v.8.2.12) [45,46], and the BI analysis was run with the MrBayes tool (v.3.2.7a) [47] in the online platform CIPRES Science Gateway (https://www.phylo.org/, accessed on 2 July 2024) [48]. The evolutionary model GTR+I+G employing 1000 non-parametric bootstrapping iterations was applied for ML analyses. For BI analysis, best-fit evolutionary models for each partitioned locus were selected based on the AIC (Akaike Information Criterion) (ITS, LSU: GTR+I+G, *rpb2*: TrN+I+G, *tef1-α*: TIM3+I+G, *tub2*: TPM2uf+I+G) and estimated using the jModelTest2 (v.2.1.6) on the XSEDE tool of CIPRES website [48]. The phylograms generated in these analyses were visualized using FigTree v.1.4.0 [49] and edited in Microsoft Office PowerPoint 2016. All the newly generated sequence data in this study were submitted to GenBank (Supplementary Table S2).

### 2.4. Morphology

Micromorphological features of isolated fungal strains (conidiophores, conidiogenous cells, and conidia) were observed and photographed using a Zeiss Axio Imager Z2 (Carl Zeiss AG, Oberkochen, Germany). Macromorphological features, such as ostioles, stromata, discs, and locules, were observed using a stereoscopic microscope (Nikon MODEL P-FMD) (Nikon Corporation, Tokyo, Japan). Sizes of at least 30 stromata, 30 discs, and 50 conidia were measured. Colony colours were recorded according to Rayner’s colour chart [50].

### 2.5. Pathogenicity Tests

To determine the pathogenicity of obtained *Cytospora* species in this study on peach trees, pathogenicity tests were conducted with two methods.

#### 2.5.1. In Vitro Inoculation

Healthy, uniform-size, 1-year-old green peach shoots were collected and the leaves were pruned; the samples were rinsed to remove the debris and surface-sterilized using 75% alcohol wipes. Using a 5 mm diameter cork-borer, holes were made on the surface of each shoot to the depth of the xylem. Randomly selected isolates from each known and novel *Cytospora* species identified in this study were used to determine pathogenicity on peach shoots. The mycelium plugs (5 mm) taken from actively grown fungal isolates were used to inoculate onto the hole made on the shoots. Each fungal isolate was inoculated onto five green shoots and five other shoots were inoculated with non-colonized PDA plugs as the control. All the inoculated shoots were planted in the pots containing the soil mixture, incubated at 25 °C and 90% relative humidity for the first 48 h, and then maintained at room temperature without humidification. Disease symptom development was evaluated on the seventh day after inoculation. Data were analyzed using Duncan’s new multiple range method by one-way analysis of variance (ANOVA) in IBM SPSS Statistics v.26.0.

#### 2.5.2. In Vivo Inoculation 

The same strains used for in vitro inoculation were employed for in vivo tests. Healthy attached trunks of two-year-old peach seedlings were surface-disinfected, and wounds were created on the trunks with a sterilized blade. Mycelium plugs taken from the fungal isolates were inoculated onto the wounds, and a piece of wet cotton was used to facilitate the humidity and covered with a parafilm. Each isolate was inoculated into three plant replicates and non-colonized PDA plugs were used as the control treatment. The pots with inoculated peach seedlings were placed in a greenhouse with constant temperature and humidity (25 °C and 90% humidity). The disease symptom development was continuously observed and recorded. Re-isolation from artificially inoculated peach seedling trunks was carried out to confirm Koch’s postulates. Fungal isolates obtained from artificially inoculated green shoots/seedlings were compared with species initially isolated by morphology analysis and sequencing the ITS region.

## 3. Results

### 3.1. Fungal Isolation

During the survey, 72 peach Cytospora canker samples were collected from five Chinese provinces. In total, 127 *Cytospora* isolates were obtained from these samples in this study (Table 2, Supplementary Table S3) and among them, 35 representative isolates were selected for further analysis.

### 3.2. Phylogenetic Analyses

**Figure 3:**
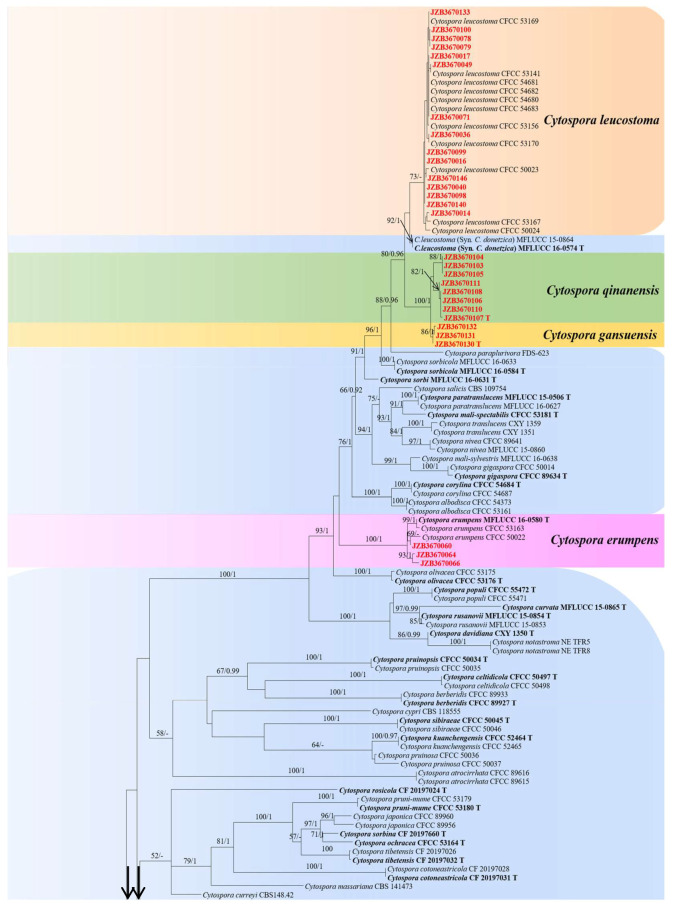

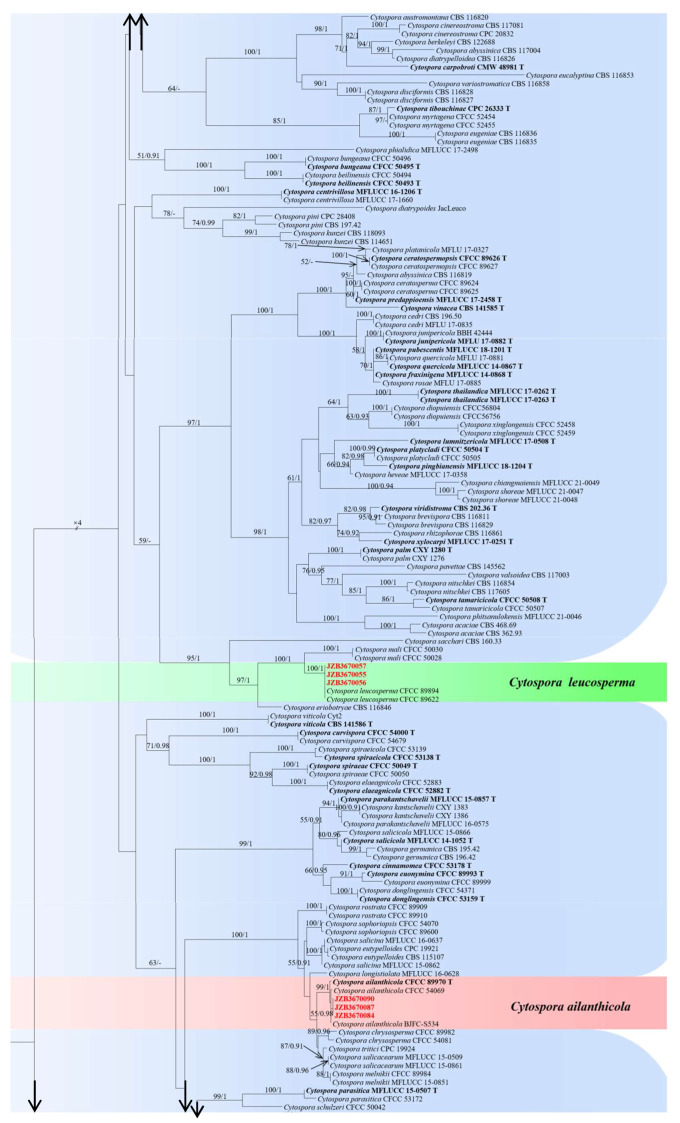

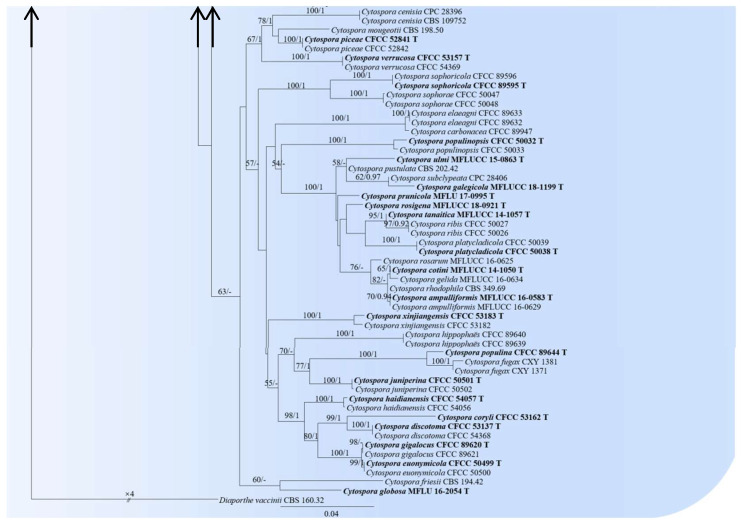
Multi-gene phylogram of *Cytospora* based on ITS-LSU-*rpb2*-*tef1-α*-*tub2* sequences. Maximum likelihood bootstrap support values above 50% and posterior probabilities above 0.90 from BI are shown near the branches, respectively. Ex-type strains are indicated in bold, and marked with “T” in the end. Isolates obtained from this study are marked in red.

The combined multi-gene sequences of the ITS-LSU-*rpb2*-*tef1-α*-*tub2* integrated phylogenetic analysis encompassed a total of 288 sequences. They consisted of 287 ingroup taxa, including 35 sequences generated from the isolates in this study, 252 reference strains obtained from the NCBI database, and one outgroup taxon *Diaporthe vaccinii* (CBS 160.32). Sequence fragments encompassed 2417 characters with gaps for a combined matrix of five genes of *Cytospora* (430 for ITS, 521 for LSU, 610 for *rpb2*, 416 for *tef1-α*, and 440 for *tub2*). For the ML analysis, the matrix included 1185 distinct alignment patterns. The model parameters were as follows: A = 0.242763, C = 0.268766, G = 0.265869, T = 0.222602; substitution rates: AC = 1.567015, AG = 4.546257, AT = 1.771439, CG = 1.136289, CT = 8.980925, GT = 1.000000; gamma distribution shape parameter α = 0.253599. The best-scoring ML tree with the final likelihood value of −41,402.886058 is given in Figure 3.

Based on the phylogenetic analyses of ITS-LSU-*rpb2*-*tef1-α*-*tub2* sequence data, the isolates obtained in this study were clustered into six separate clades (Figure 3). A similar tree topology was observed in both ML and BI analyses. The isolates obtained in this study were identified as four known *Cytospora* species, *C. erumpens*, *C. ailanthicola*, *C. leucosperma*, and *C. leucostoma*, and two novel species; named *C. gansuensis* sp. nov. and *C. qinanensis* sp. nov.

### 3.3. Taxonomy

**Figure 4:**
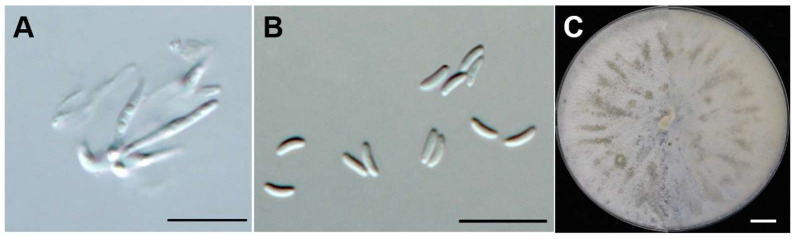
Morphological characteristics of *C. ailanthicola* (JZB3670084). (**A**) Conidiophores. (**B**) Conidia. (**C**) Upper and reverse view of the 7-day-old colony on PDA. Scale bars: (**A**,**B**) = 10 μm, (**C**) = 1 cm.

***Cytospora ailanthicola*** X.L. Fan & C.M. Tian, Persoonia 45: 13, 2020 [51] (Figure 4).

MycoBank number: MB830146.

Asexual morph: On peach branches. Conidia are hyaline, transparent, unicellate, allantoid, smooth, aseptate, thin-walled, 3.16–3.86 × 0.79–1.18 μm (average = 3.55 × 1.02 μm, n = 50). Sexual morph: not observed.

Culture characteristics: On PDA medium, grow 9 cm diam. after five days. The initial colony is white, with dense hyphae and radiative growth. Later, the colony colour is beige and forms randomly distributed pycnidium on the colony surface.

Material examined: China, Beijing, Pinggu Districts, on peach branches, May 2020, Ying Zhou, dry cultures: JZBH3670087, JZBH3670090, living cultures: JZB3670087, JZB3670090; China, Beijing, Pinggu Districts, on a peach branch, March 2021, Ying Zhou, dry cultures: JZBH3670084, living cultures JZB3670084.

Additional materials: see Supplementary Table S3.

Notes: *Cytospora ailanthicola* was first introduced by Fan et al. [51] as causing canker disease in *Ailanthus altissima* in China. In this study, based on morpho-molecular data, we identified seven isolates that resemble *C. ailanthicola*. Our isolates share similar morphology with the type species of *C. ailanthicola* (CFCC 89970); however, our isolates (3.55 × 1.02 μm) have smaller conidia than type species (4.5 × 1.5 μm). Here, we first report the *C. ailanthicola* associated with branches of *Prunus persica* in the world. According to our pathogenicity test results, this species did not exhibit any disease symptom development on the tested peach shoots or trunks.

**Figure 5:**
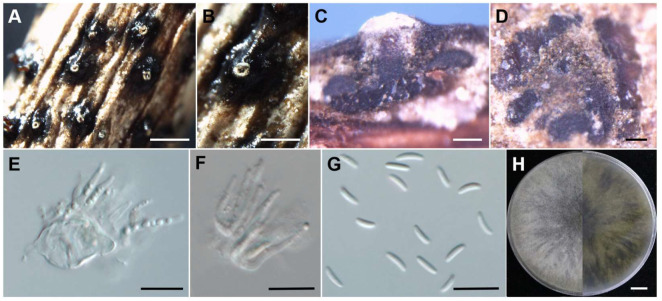
Morphological characteristics of *C. erumpens* (JZB3670059). (**A**,**B**) Asexual stroma. (**C**) Longitudinal section of asexual stroma. (**D**) Cross section of asexual stroma. (**E**,**F**) Conidiophore. (**G**) Conidia. (**H**) Upper and reverse view of the 7-day-old colony on PDA. Scale bars: (**A**) = 1 mm, (**B**) = 500 μm, (**C**,**D**) = 100 μm, (**E**–**G**) = 10 μm, (**H**) = 1 cm.

***Cytospora erumpens*** Norph. et al., Mycosphere 8: 64, 2017 [29] (Figure 5).

MycoBank number: MB552604.

Asexual morph: On peach branches, Pycnidial stromata ostiolate, Black fruiting bodies immersed in the bark. The stromata slightly protrude, round and black on the outer edge, with multiple locules, and shared walls in a radial pattern with a diameter of 623–977 μm (average = 803 μm, n = 30). The ostiole is black and round, with one ostiole per disc, and a diameter of 52–141 μm (average = 89 μm, n = 30). Conidia are hyaline, unicellate, allantoid, smooth, aseptate, thin-walled, 5.64–7.23 × 1.41–1.71 μm (average = 6.18 × 1.52 μm, n = 50). Sexual morph: not observed.

Culture morphology: On PDA medium, grow 9 cm in diameter after five days. It is white in the initial stage; the hyphae are dense, and later, they become yellow-grey and covered with white villi. The colonies are flat, and there is no pycnidium observed on the surface.

Material examined: China, Jilin Province, Jiaohe City, on peach branches, 17 July 2022, Zhizheng He and Ying Zhou, dry cultures: JZBH3670060, JZBH3670064, JZBH3670066, living cultures JZB3670060, JZB3670064, JZB3670066.

Additional materials: see Supplementary Table S3.

Notes: In this study, we obtained 12 isolates and identified them as *C. erumpens* using morphological and phylogenetic analysis data. This species was first described on the dead branches of *Salix* in Russia [29]. Our isolates share similar morphology with the type species of *C. erumpens* (MFLUCC 16-0580); however, our isolates (6.18 × 1.52 μm) have smaller conidia than type species (6.4 × 1.4 μm). Here, we also report the first host association of *C. erumpens* on *Prunus persica* in the world.

**Figure 6:**
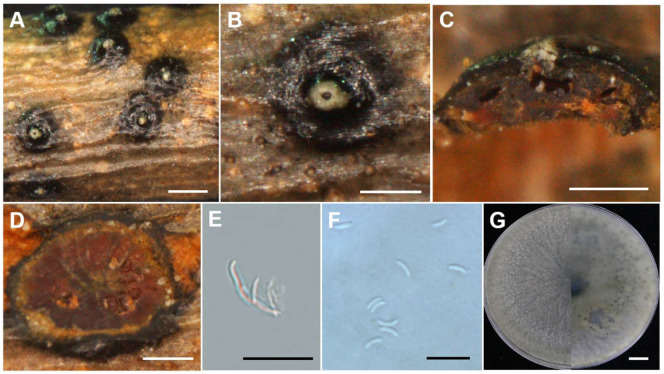
Morphological characteristics of *C. gansuensis* sp. nov (Holotype JZB3670130). (**A**,**B**) Asexual stroma. (**C**) Longitudinal section of asexual stroma. (**D**) Cross section of asexual stroma. (**E**) Conidiophore. (**F**) Conidia. (**G**) Upper and reverse view of the 7-day-old colony on PDA. Scale bars: (**A**) = 1 mm, (**B**–**D**) = 500 μm, (**E**,**F**) = 10 μm, (**G**) = 1 cm.

***Cytospora gansuensis*** Z. Z. He & Y. Zhou, sp. nov. (Figure 6).

Mycobank number: MB855530.

Etymology: the name reflects the location where the holotype was collected: Gansu Province, China.

Holotype: JZB3670130.

Asexual morph: On peach branches, Pycnidial stromata ostiolate, immersed in bark, with black conceptacle. The stromata slightly protrude, are round, and black on the outer edge, with multiple locules and shared walls, forming a radiant structure with a diameter of 828–1716 μm (average = 1189 μm, n = 30). The ostiole is black and round, with one ostiole per disc, and a diameter of 66–142 μm (average = 100 μm, n = 30). Conidia are hyaline, transparent, unicellate, allantoid, eguttulate, smooth, aseptate, thin-walled, 4.09–6.11 × 1.03–1.47 μm (average = 5.03 × 1.27 μm, n = 50). Sexual morph: not observed.

Culture morphology: On PDA medium, grow to 9 cm in diameter after five days. It is white in the initial stage, with dense hyphae and felt-like colonies. In the later stage, melanin is produced, which gradually turns dusty grey and is covered by white villi. Small conidiomata are randomly distributed on the surface of the colony and densely distributed.

Material examined: China, Gansu Province, Qin’an County, on peach branches, 2 May 2023, Gan Wang, Holotype: JZBH3670130, ex-type living culture: JZB3670130.

Additional materials: see Supplementary Table S3.

Note: In the present phylogenetic analysis, we identified three isolates that were producing separate sister branches to a known *Cytospora* species, *C. leucostoma* (Syn. *C. donetzia*), and one novel species introduced in this study (*C. qinanensis* sp. nov.). Based on morphological and phylogenetic data, here, we treated these isolates (JZB3670130–JZB3670132) as a new species, named *C. gansuensis* sp. nov. Morphologically, *C. leucostoma* and *C. qinanensis* sp. nov. can be differentiated with our new species by their locules, ostioles, and conidial size. Our isolates produce locules smaller (1189 μm) than *C. leucostoma* (1310 μm) and bigger than the ones produced by *C. qinanensis* sp. nov. (1053 μm). Our species produce smaller ostioles (100 μm) than *C. leucostoma* (129 μm) and *C. qinanensis* (114 μm). Further, the conidia produced by this new species (*C. gansuensis* sp. nov) are smaller (5.03 × 1.27 μm) than *C. leucostoma* (5.54 × 1.49 µm) and bigger than the ones produced by *C. qinanensis* sp. nov (4.88 × 1.17 μm). The nucleotide differences between the holotype strain of *C. gansuensis* sp. nov (JZB3670130) and *C. leucostoma* (CFCC 50023) are ITS: 2.22% (13/586 bp), LSU: 1.35% (7/519 bp), *rpb2*: 1.51% (11/727 bp), *tef1-α* 4.13% (25/605 bp), and *tub2*: 1.40% (9/644 bp). The nucleotide differences between the holotype strain of *C. gansuensis* sp. nov. (JZB3670130) and *C. qinanensis* (JZB3670107) are ITS: 1.37% (8/586 bp), LSU: 1.35% (1/519 bp), *rpb2*: 1.51% (11/727 bp), *tef1-α* 2.15% (13/605 bp), and *tub2*: 1.40% (9/644 bp).

**Figure 7:**
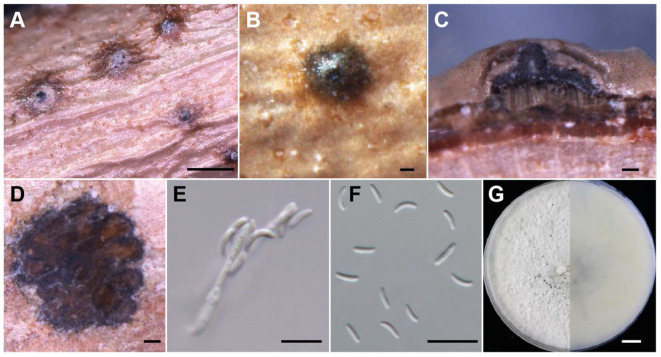
Morphological characteristics of *C. leucosperma* (JZB3670055). (**A**,**B**) Asexual stroma. (**C**) Longitudinal section of asexual stroma. (**D**) Cross section of asexual stroma. (**E**) Conidiophore. (**F**) Conidia. (**G**) Upper and reverse view of 7-day-old colony on PDA. Scale bars: (**A**) = 1 mm, (**B**–**D**) = 100 μm, (**E**,**F**) = 10 μm, (**G**) = 1 cm.

***Cytospora leucosperma*** (Pers.) Fr., Syst. Mycol. 2: 543, 1823 [52] (Figure 7).

MycoBank number: MB213600.

Asexual morph: On peach branches, Pycnidial stromata ostiolate, immersed in bark, without black conceptacle; the stromata slightly protrude to flatten through the bark surface, with multiple locules, shared walls and are arranged in a circular pattern with a diameter of 510–905 m (average = 639 µm, n = 30). The ostiole is black and round, with one ostiole per disc, and a diameter of 55–129 µm (average = 107 µm, n = 30). Conidia are hyaline, transparent, unicellate, allantoid, eguttulate, smooth, aseptate, thin-walled, 3.70–4.85 × 1.05–1.53 µm (average = 4.57 × 1.27 µm, n = 50). Sexual morph: not observed.

Culture characteristics: On PDA medium, colonies grow quickly, reaching 9 cm in diameter after three days. In the initial stage, it appears white, with dense hyphae and radiating growth. In the later stages, it turns cream, and the colonies form randomly distributed pycnidium on the surface of the colonies.

Material examined: China, Beijing, Shunyi Districts, on peach branches, 1 November 2022, Zhizheng He and Ying Zhou, dry cultures: JZBH3670055-JZBH3670057, living cultures JZB3670055-JZB3670057.

Notes: In this study, we obtained three *Cytospora* isolates and they were identified as *C. leucosperma*. Our isolates share similar morphology with the *C. leucosperma* in Fan et al.’s study [51]; however, the conidia of our isolates (3.70–4.85 × 1.05–1.53 μm) were a little smaller than type species (3.5–5.5 × 1–1.5 μm).

**Figure 8:**
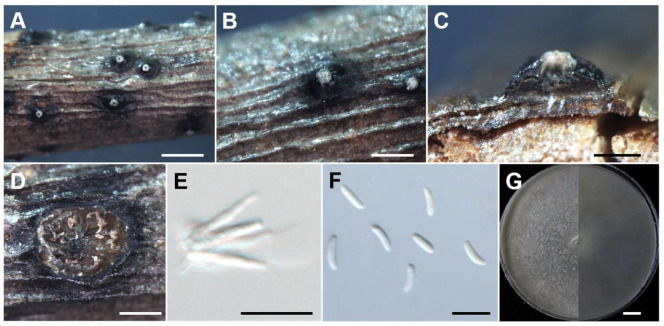
Morphological characteristics of *C. leucostoma* (JZB3670075). (**A**,**B**) Asexual stroma. (**C**) Longitudinal section of asexual stroma. (**D**) Cross section of asexual stroma. (**E**) Conidiophore. (**F**) Conidia. (**G**) Upper and reverse view of the 7-day-old colony on PDA. Scale bars: (**A**,**B**) = 1 mm, (**C**,**D**) = 500 μm, (**E**,**F**) = 10 μm, (**G**) = 1 cm.

***Cytospora leucostoma*** (Pers.) Sacc., Michelia 2: 264, 1881 [53] (Figure 8).

MycoBank number: MB213669.

Asexual morph: On peach branches, Pycnidial stromata ostiolate, immersed in bark, with black conceptacle. The stromata slightly protrude, are round, and black on the outer edge. Locules are numerous, subdivided frequently by invaginations with independent walls, and with a diameter of 922–1674 μm (average = 1310 μm, n = 30). The ostiole is black and round, with one ostiole per disc, and a diameter of 78–178 μm (average = 129 μm, n = 30). Conidia are hyaline, transparent, unicellate, allantoid, eguttulate, smooth, aseptate, thin-walled, 5.11–7.06 × 1.40–1.63 μm (average = 5.54 × 1.49 μm, n = 50). Sexual morph: not observed.

Culture morphology: On PDA medium, grow 9 cm diam. after five days. It is white in the initial stage, with dense hyphae. In the later stage, it is blue-grey and covered with white fluff, and randomly distributed pycnidium is formed on the colony surface.

Material examined: China, Liaoning Province, Dalian City, on peach branches, 11 November 2022, Zhizheng He and Ying Zhou, dry cultures: JZBH3670071, JZBH3670078, JZBH3670079, living cultures JZB3670071, JZB3670078, JZB3670079; China, Beijing, Shunyi Districts, on peach branches, 1 November 2022, Zhizheng He and Ying Zhou, dry cultures: JZBH3670036, JZBH3670040, JZBH3670049, living cultures JZB3670036, JZB3670040, JZB3670049; China, Beijing, Pinggu Districts, on peach branches, 21 September 2022, Ying Zhou, dry cultures: JZBH3670014, JZBH3670016, JZBH3670017, living cultures JZB3670014, JZB3670016, JZB3670017; China, Gansu Province, Qin’an City, on peach branches, 2 May 2023, Gan Wang, dry cultures: JZBH3670098–JZBH3670100, living cultures JZB3670098–JZB3670100; China, Shandong Province, Jinan City, on peach branches, 22 May 2023, Zhizheng He and Ying Zhou, dry cultures: JZBH3670133, JZBH3670140, JZBH3670146, living cultures JZB3670133, JZB3670140, JZB3670146.

Additional materials: see Supplementary Table S3.

Notes: *Cytospora leucostoma* is a common species causing canker disease of Rosaceae hosts, especially Prunoideae in China [54,55,56,57]. In this study, we obtained 93 isolates and identified them as *C. leucostoma* using morphological and phylogenetic analysis data. This species was the most widely distributed; we obtained it from all the provinces in this study except Jilin. Our isolates share similar morphology with the *C. leucostoma* isolate (CFCC 50023) [51]; however, our isolates (5.11–7.06 × 1.40–1.63 μm) have bigger conidia than type species (4.5–5.5 × 1–1.5 μm).

**Figure 9:**
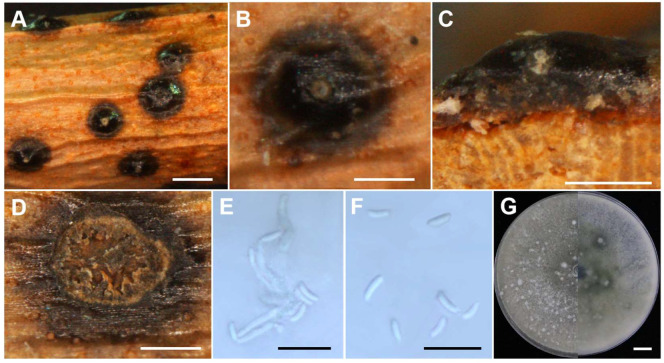
Morphological characteristics of *C. qinanensis* sp. nov. (holotype JZB3670107). (**A**,**B**) Asexual stroma. (**C**) Longitudinal section of asexual stroma. (**D**) Cross section of asexual stroma. (**E**) Conidiophore. (**F**) Conidia. (**G**) Upper and reverse view of 7-day-old colony on PDA. Scale bars, (**A**) = 1 mm, (**B**–**D**) = 500 μm, (**E**,**F**) = 10 μm, (**G**) = 1 cm.

***Cytospora qinanensis*** Z. Z. He & Y. Zhou, sp. nov. (Figure 9).

Mycobank number: MB855529.

Etymology: the name reflects the location where the holotype was collected, Qin’an County, Gansu Province, China.

Holotype: JZB3670107.

Asexual morph: On peach branches, Pycnidial stromata ostiolate, immersed in bark, with black conceptacle. The stromata slightly protrude, are round, and black on the outer edge, with multiple locules and shared walls, forming an irregular shape structure with a diameter of 738–1236 μm (average = 1053 μm, n = 30). The ostiole is black and round, with one ostiole per disc, and a diameter of 80–178 μm (average = 114 μm, n = 30). Conidia are hyaline, transparent, unicellate, allantoid, eguttulate, smooth, aseptate, thin-walled, 4.58–5.16 × 0.93–1.42 μm (average = 4.88 × 1.17 μm, n = 50). Sexual morph: not observed.

Culture morphology: On PDA medium, grow 9 cm diam. after five days. It is white in the initial stage, with dense hyphae. In the later stage, melanin is produced, gradually turning mouse grey and covered by white villi, on which a conidium randomly distributed on the surface of the colony is formed. The pycnidium is large, and the surface is covered by white villi. In the later stage, the top of the pycnidium discharges hyaline spore masses and later becomes brownish-red.

Material examined: China, Gansu Province, Qin’an County, on peach branches, 2 May 2023, Gan Wang, Holotype: JZBH3670107, ex-type living culture: JZB3670107.

Additional materials: see Supplementary Table S3. 

Note: In this present phylogenetic analysis, we identified nine isolates produced separate sister branches to the other known *Cytospora* species; *C. leucostoma* (Syn. *C. donetzia*), and here we treated these isolates (JZB3670103–JZB3670111) as a new species-based on morphological and phylogenetic data. Morphologically, our novel species, *Cytospora qinanensis* sp. nov., can be distinguished by their locules, ostioles, and conidial size, with *C. leucostoma* producing smaller locules (1053 μm to 1310 μm, respectively), smaller ostioles (114 μm to 129 μm, respectively), and smaller conidia (4.88 × 1.17 μm to 5.54 × 1.49 µm, respectively). Fan et al. [51] suggest that *C. donetzica* should be synonymized with *C. leucostoma* based on currently available DNA data and morphological similarities. The nucleotide differences between holotype species of *C. qinanensis* sp. nov. (JZB3670107) and *C. leucostoma* (CFCC 50023) are ITS: 2.56% (15/587 bp), LSU: 1.54% (8/519 bp), *rpb2*: 1.65% (12/727 bp), *tef1-α* 5.29% (32/605 bp), and *tub2*: 1.40% (9/644 bp).

### 3.4. Pathogenicity Test

#### 3.4.1. In Vitro Inoculation

The symptom development started 2 days after the inoculation. On the second day of inoculation, there was a water-stained lesion at the inoculation site, and in the following cases, glue flow occurred (gummosis), and with the decrease in humidity, the lesion showed a brown, round pattern. On some of the more severely diseased branches, black, punctate, raised fruiting bodies began to appear gradually from the fifth day after inoculation.

**Figure 10:**
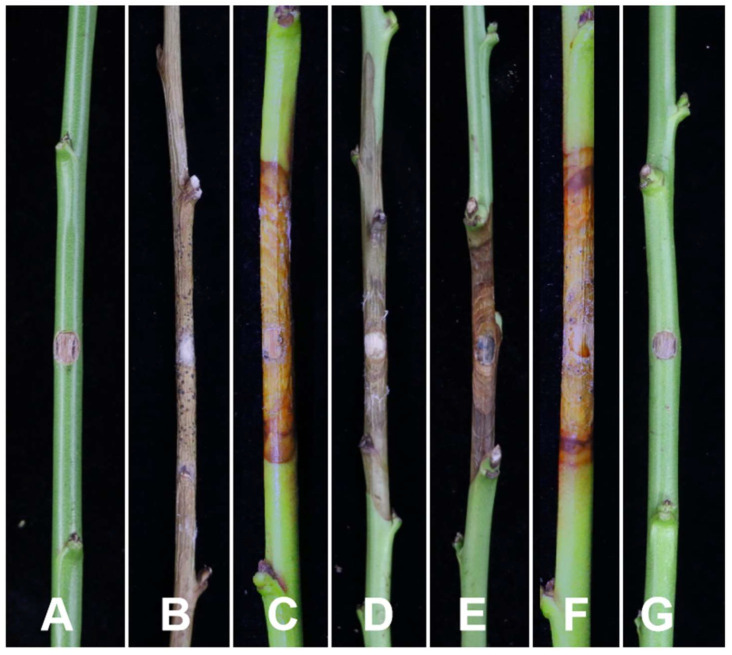
Disease symptom development on peach shoots after seven days of inoculation. (**A**) *C. ailanthicola* (JZB3670084). (**B**) *C. erumpens* (JZB3670059). (**C**) *C. gansuensis* sp. nov (JZB3670132). (**D**) *C. leucosperma* (JZB3670057). (**E**) *C. leucostoma* (JZB3670023). (**F**) *C. qinanensis* sp. nov (JZB3670103). (**G**) Control.

**Figure 11:**
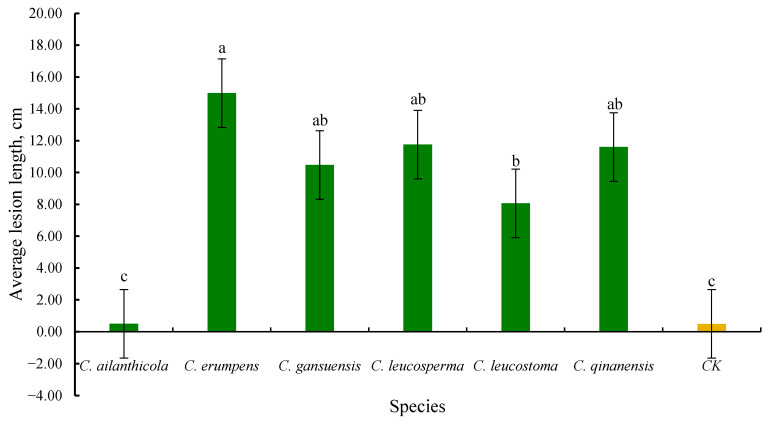
Average lesion length (cm) resulting from inoculation trials on *Prunus persica* shoots after the seventh day of inoculation. Vertical bars represent the standard error of means. Different letters above the bars indicate treatments that were significantly different (*p* = 0.05).

The lesion length was measured on the seventh day after inoculation and the symptoms and the average lesion lengths are shown below (Figure 10 and Figure 11).

According to the above pathogenicity experiment data, green peach shoots inoculated with *C. leucostoma*, *C. erumpens*, *C. leucosperma*, *C. qinanensis* sp. nov., and *C. gansuensis* sp. nov., isolates developed symptoms. Among them, *C. erumpens* produces the highest lesion length, followed by *C. gansuensis* sp. nov., *C. leucosperma*, and *C. qinanensis* sp. nov., while *C. leucostoma* has the least lesion length, respectively. During this experiment, *C. ailanthicola* and control shoots did not produce any symptoms on peach shoots (Figure 10 and Figure 11).

#### 3.4.2. In Vivo Inoculation

**Figure 12:**
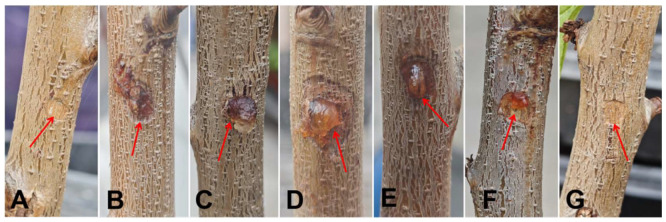
Disease symptoms observed on the peach trunks after one month of in vivo inoculation. (**A**) *C. ailanthicola* (JZB3670084). (**B**) *C. erumpens* (JZB3670059). (**C**) *C. gansuensis* sp. nov. (JZB3670132). (**D**) *C. leucosperma* (JZB3670055). (**E**) *C. leucostoma* (JZB3670023). (**F**) *C. qinanensis* sp. nov. (JZB3670103). (**G**) Control. The inoculation site is marked with a red arrow.

Six isolates of *Cytospora* species recovered from this study were inoculated onto peach trunks. However, among them, plants inoculated with *C. leucostoma*, *C. erumpens*, *C. leucosperma*, *C. qinanensis* sp. nov., and *C. gansuensis* sp. nov only showed varying degrees of gummosis at the wound site, while *C. ailanthicola* and control treatments did not show any symptom development. After 20 days of inoculation, mild infection symptoms appeared (including gumming and the lesion becoming darker), and the lesion spread was slow. One month after inoculation, symptoms were consistent with those observed in the field, including cortical decay, slightly concave diseased areas, and dark brown lesions. Later, black punctate, raised fruiting bodies were observed on several trunks. Neither plants inoculated with *C. ailanthicola* nor the control plants exhibited any typical lesions or gummosis throughout the experimental period (Figure 12).

## 4. Discussion

This study represents the first comprehensive characterization of species in *Cytospora* isolated from peach trees with Cytospora canker symptoms in northern China. Based on multi-loci phylogenetic analyses and morphological characteristics, 127 *Cytospora* isolates were classified into four known and two novel species. These species include *C. ailanthicola*, *C. erumpens*, *C. leucosperma* and *C. leucostoma* and the new species described here, namely, *C. gansuensis* sp. nov. and *C. qinanensis* sp. nov. Of the four known species reported here, *C. erumpens* was reported on peach for the first time. The isolation frequency of *Cytospora* species is as follows: *C. leucostoma* 73.60%, *C. erumpens* 9.60%, *C. qinanensis* sp. nov., 6.40%, *C. ailanthicola* 5.60%, *C. leucosperma* 2.40%, and *C. gansuensis* sp. nov., 2.40%. In this study, *Cytospora leucostoma* is the most common and widely distributed species and it was isolated from all provinces except Jilin. *Cytospora erumpens* was isolated only from the samples collected in Jilin and *C. leucosperma* and *C. ailanthicola* were only isolated from samples in Beijing. The novel *Cytospora* species, *C. gansuensis* sp. nov. and *C. qinanensis* sp. nov. were found only in Gansu Province.

Species of *Cytospora* are mainly known as important plant pathogens causing diebacks and cankers on a wide range of host plants worldwide [29]. The delineation of *Cytospora* species is difficult due to the similar morphological features, unavailability of sequence data for type species, and poor understanding of phylogeny [29,58]. Initially, the classification of *Cytospora* was based on the number and arrangement of its pycnidium and perithecia [59]. However, in 1935, Défago questioned the usefulness of morphological characteristics in defining *Cytospora* species [60]. In 1985, Spielman reported that the imperfect state of *C. leucosperma* was challenging to differentiate from other species of *Cytospora* [61]. Due to the phenotypic plasticity of *Cytospora*, it is difficult to differentiate species solely based on morphology [62]. Previously, due to overlapping morphological features, several studies have also relied on host association [63]. However, with continuous research on *Cytospora* species, many scholars have determined that *Cytospora* can occur across multiple hosts. On the same host, various symptoms can be exhibited and multiple *Cytospora* fungi can co-exist, resulting in a relatively chaotic situation in the classification of *Cytospora* in the early stages [63,64,65,66,67,68,69].

Molecular systematics offers an improved approach to the classification of *Cytospora* fungi [29,70]. It is stated that specific gene sequences provide a standardized method for genetic material-based biological identification, updating criteria for species identification [71,72]. The universal barcode of fungi includes the internal transcription spacer region (ITS) [73]. However, the ITS region lacks robust discriminative ability and cannot identify genera with low intraspecific variation [74,75]. Therefore, more genetic information may be needed to achieve accurate identification of species like *Cytospora*, such as the secondary region of protein-coding gene loci, including transcription elongation factor (*tef1-α*), Beta-tubulin (*tub*), and others [76]. In this study, we also utilize combined sequence data of five gene regions, ITS-LSU-*rpb2*-*tef1-α*-*tub2,* and provided an updated phylogenetic tree for the *Cytospora* genus (Figure 3).

The results of the pathogenicity experiments indicate that except for *C. ailanthicola*, all other *Cytospora* species obtained in this study are pathogenic to the tested *Prunus persica* shoots in vitro and trunks in vivo. *Cytospora leucostoma* is the most common pathogen causing Cytospora canker in stone fruit trees worldwide, and it is estimated that this pathogen causes 15–20% annual yield loss in peach production in Colorado, USA [11,77]. *Cytospora leucostoma* was first discovered by Maneval [78] in the United States and showed pathogenicity to *Pyrus* and *Prunus* plants, including peaches and apricots. Further, this species was subsequently found to also infect various plants like *Malus* [79] and *Sorbus* [80]. In China, this species is the most common cause of stem cankers on woody Rosaceae hosts [51,81]. In this study, the pathogenicity test results revealed that, compared to other *Cytospora* isolates (*C. erumpens*, *C. gansuensis* sp. nov, *C. leucosperma*, and *C. qinanensis* sp. nov), *C. leucostoma* was not aggressive and produced the shortest lesion length on peach shoots (Figure 11).

In this study, we found that *C. leucosperma* has a strong pathogenicity on the tested peach shoots and trunks. This species was previously named *Valsa ambiens*, and it was first identified in the United States [82]. It showed pathogenicity to various plants, including *Crataegus mollis*, *Rubus strigosus*, *Ulmus americana* [82], *Prunus* [83], *Malus* [79], *Populus*, *Salix* [84], and others. This species was first reported on peach trees by Alfieri et al. [85] in the United States, and the first domestic case of *C. leucosperma* on peach was reported in Xinjiang, China [86]. Interestingly, two new species discovered in this study, *C. qinanensis* sp. nov. and *C. gansuensis* sp. nov., also showed similar pathogenicity to the peach shoots and trunks as *C. leucosperma*. Therefore, it is important to further study whether these two species also have a host range as wide as *C. leucosperma* or not. The pathogenic potential and co-infection of these two new species with *C. leucosperma* need to be confirmed by further studies. Additional studies are recommended to evaluate the cross-infection potential of *C. qinanensis* sp. nov. and *C. gansuensis* sp. nov. on other Rosaceae hosts as well.

*Cytospora erumpens* showed high aggressiveness on peach shoots in this study and this species was initially identified to be pathogenic to *Salix fragilis* in Russia [29]. So far, this species has only been found on *Prunus padus* and *Salix fragilis* [51]. Thus, in this study, we reported for the first time the association of *C. erumpens* with a peach host causing Cytospora canker disease. Further study of the pathogenic potential of this species on other stone fruits is also needed.

*Cytospora ailanthicola* was first found on *Ailanthus altissima* [51]. Later, this species was identified as causing cankers on *Cerasus serrulate* (cherry blossom) and *Salix matsudana* (Willow) in China [87,88,89]. In this study, we also reported the first host association of *C. ailanthicola* with *Prunus persica*. Further, we found that *C. ailanthicola* is not pathogenic to the peach shoots and trunks tested, but it may potentially act as an endophytic or saprophytic fungus. However, further studies are recommended to confirm its existence in peach orchards in China.

In the in vivo inoculation experiment, the five *Cytospora* species exhibited different degrees of gummosis development in the wound after inoculation. *Cytospora ailanthicola* and the control plants did not show any evidence of gummosis development. During our field survey, we also observed gummosis on the peach trees exhibiting Cytospora canker disease and we also isolated *Cytospora* fungi from branches with peach gummosis. Peach gummosis can be separated into infectious gummosis and non-infectious gummosis [90]. Infectious gummosis is mainly a pathological disease caused by the infection of pathogens, mainly *Botryosphaeria* spp. [90,91]; in addition to that, *Lasiodiplodia theobromae*, *Pestalotiopsis disseminata*, *Neofusicoccum parvum* and *Fusarium lateritium* can also cause peach gummosis [92,93,94]. Non-infectious gummosis is a physiological gummosis, mainly caused by non-biological factors, such as sunburn, freezing injury, plant diseases, insect pests, and man-made mechanical damage [95]. In our study, the result of the in vivo experiment (the gummosis we observed) may be due to physiological gummosis caused by the stimulation of wounds by metabolites produced during the invasion of inoculated pathogenic fungi.

Generally, peach Cytospora canker is thought to be caused only by *C. leucostoma* [96]. However, in our study, we have identified several species, including two novel *Cytospora* species, that can cause peach Cytospora canker in China. The results obtained in this study apply only to samples collected at collection sites located in northern China. Therefore, we recommend that subsequent sampling is expanded to investigate the prevalence of *Cytospora* species in other peach-producing areas in China. Further, this study provided baseline data to prevent and control peach Cytospora canker in China and we recommend further research to elucidate the impact of the novel pathogenic fungi identified in this study on Chinese peach cultivation.

## Figures and Tables

**Table 1:** Primers and PCR protocols used in this study.

Genes	Primers	Primer Sequence	Annealing Temperature (°C)	References
ITS	ITS4	5′-TCCTCCGCTTATTGATATGC-3′	58	White et al. 1990 [38]
	ITS5	5′-GGAAGTAAAAGTCGTAACAAGG-3′		
LSU	LR0R	5′-GTACCCGCTGAACTTAAGC-3′	56	Vilgalys and Hester 1990 [39]
	LR5	5′-TCCTGAGGGAAACTTCG-3′		
*rpb2*	RPB2-5F2	5′-GGGGWGAYCAGAAGAAGGC-3′	54	Sung et al. 2007 [40];Liu et al. 1999 [41]
	RPB2-7cR	5′-CCCATRGCTTGYTTRCCCAT-3′		
*tef1-α*	EF1-688F	5′-CGGTCACTTGATCTACAAGTGC-3′	56	Alves et al. 2008 [42]
	EF1-1251R	5′-CCTCGAACTCACCAGTACCG-3′		
*tub2*	BT2F	5′-GTBCACCTYCARACCGCYCARTG-3′	54	Woudenberg et al. 2009 [43]
	BT4R	5′-CCRGAYTGRCCRAARACRAAGTTGTC-3′		

**Table 2:** Sample collection and isolation status of *Cytospora*.

Location of Sample Collection	Number of Samples	Number of Isolates Obtained
Pinggu, Beijing	15	23
Shunyi, Beijing	10	24
Qin’an, Gansu	20	25
Jiaohe, Jilin	7	12
Huludao and Dalian, Liaoning	11	25
Jinan, Shandong	9	18
Total	72	127

## Data Availability

The original contributions presented in this study are included in the article and Supplementary Material. Further inquiries can be directed to the corresponding authors.

## Supplementary Materials

The following supporting information can be downloaded at: https://www.mdpi.com/article/10.3390/jof10120843/s1, Table S1. GenBank accession numbers of the sequences used for phylogenetic analysis in this study. Table S2. Isolates and GenBank accession numbers of sequences obtained from this study. Table S3. Isolates obtained in this study.
