# Supplementary material for: Diversity of Cytospora Species Associated with Trunk Diseases of Prunus persica (Peach) in Northern China
Source: J Fungi (Basel). 2024 Dec 5;10(12):843. doi: 10.3390/jof10120843 (PMC11678414; doi:10.3390/jof10120843)
Supplement: Supplementary file 1 [file jof-10-00843-s001.zip › Supplementary Materials-Table S1.pdf]

Table S1. GenBank accession numbers of the sequences used for phylogenetic analysis in this study

Species	Strain number	Host (Latin name)	Location	GenBank Accession number				
				ITS	LSU	*rpb2*	*tef1-α*	*tub2*
Cytospora. abietis	CBS 185.42	*Abies alba*	Switzerland	KY051893	KX965352	NA	NA	NA
	CPC 28394	*Abies alba*	Switzerland	KY051982	NA	KX965581	KX965222	KX965029
C. abyssinica	CBS 117004	*Eucalyptus saligna*	Ethiopia	KY051833	KX965299	KX965495	KX965098	NA
	CBS 116819	*Eucalyptus globulus*	Ethiopia	KY051794	KX965257	NA	KX965067	NA
C. acaciae	CBS 468.69	*Ceratonia siliqua*	Spain	KY051937	KX965394	NA	KX965181	KX964990
	CBS 362.93	*Quercus ilex*	Italy	KY051929	KX965386	KX965547	KX965173	KX964983
C. ailanthicola	CFCC 89970T*	*Ailanthus altissima*	Ningxia, China	MH933618	MH933653	MH933592	MH933494	MH933565
	BJFC-S534	*Populus alba* var.*pyramidalis*	Ningxia, China	OK147832	NA	NA	NA	OK303700
	CFCC 54069	*Populus tomentosa*	Beijing, China	MZ702622	NA	OK303543	OK303604	OK303671
C. albodisca	CFCC 54373	*Platycladus orientalis*	Beijing, China	MW418407	MW418419	MW422910	MW422922	MW422934
C. ampulliformis	MFLUCC 16-0583T	*Sorbus intermedia*	Russia	KY417726	KY417760	KY417794	NA	NA
	MFLUCC 16-0629	*Acer platanoides*	Russia	KY417727	KY417761	KY417795	NA	NA
C. atrocirrhata	CFCC 89615	*Juglans regia*	Qinghai, China	KR045618	KR045700	KU710946	KP310858	KR045659
	CFCC 89616	*Juglans regia*	Qinghai, China	KR045619	KR045701	KU710947	KP310859	KR045660
C. austromontana	CBS 116820	*Eucalyptus pauciflora*	New South Wales	KY051795	KX965258	KX965472	NA	NA
C. beilinensis	CFCC 50493T	*Pinus armandii*	Beijing, China	MH933619	MH933654	NA	MH933495	MH933561
	CFCC 50494	*Pinus armandii*	Beijing, China	MH933620	MH933655	NA	MH933496	MH933562
C. berberidis	CFCC 89927T	*Berberis dasystachya*	Qinghai, China	KR045620	KR045702	KU710948	KU710913	KR045661
	CFCC 89933	*Berberis dasystachya*	Qinghai, China	KR045621	KR045703	KU710949	KU710914	KR045662
C. berkeleyi	CBS 122688	*Abies alba*	New York, US	KY051877	KX965342	KX965522	KX965134	KX964949
C. brevispora	CBS 116811	*Eucalyptus grandis x tereticornis*	Congo	KY051786	KX965250	KX965468	KX965060	KX964896

* T = model strain, the same below.

	CBS 116829	*Eucalyptus camaldulensis*	Venezuela	KY051803	KX965267	KX965477	KX965073	KX964909
C. bungeana	CFCC 50495[T]	*Pinus bungeana*	Shanxi, China	MH933621	MH933656	MH933593	MH933497	MH933563
	CFCC 50496	*Pinus bungeana*	Shanxi, China	MH933622	MH933657	MH933594	MH933498	MH933564
C. carbonacea	CFCC 89947	*Ulmus pumila*	Qinghai, China	KR045622	KP310812	KU710950	KP310855	KP310825
C. carpobroti	CMW 48981[T]	*Carpobrotusedulis*	South Africa	MH382812	MH411216	NA	MH411212	MH411207
C. cedri	CBS 196.50	*Abies alba*	Italy	KY051905	KX965364	KX965534	KX965153	NA
	MFLU 17-0835	*Rubus sp.*	Italy	MN871816	MN873004	MN871989	NA	NA
C. celtidicola	CFCC 50497[T]	*Celtis sinensis*	Anhui, China	MH933623	MH933658	MH933595	MH933499	MH933566
	CFCC 50498	*Celtis sinensis*	Anhui, China	MH933624	MH933659	MH933596	MH933500	MH933567
C. cenisia	CBS 109752	*Juniperus communis*	Austria	KY051771	KX965236	KX965461	KX965050	KX964883
	CPC 28396	NA	NA	KY051983	NA	KX965582	KX965223	KX965030
C. centrivillosa	MFLUCC 16-1206[T]	*Sorbus domestica*	Italy	MF190122	MF190068	MF377601	NA	NA
	MFLUCC 17-1660	*Sorbus domestica*	Italy	MF190123	MF190069	MF377600	NA	NA
C. ceratosperma	CFCC 89624	*Juglans regia*	Gansu, China	KR045645	KR045724	KU710976	KP310860	KR045686
	CFCC 89625	*Juglans regia*	Gansu, China	KR045646	KR045725	KU710977	KP310861	KR045687
C. ceratospermopsis	CFCC 89626[T]	*Juglans regia*	Shannxi, China	KR045647	KR045726	KU710978	KU710934	KR045688
	CFCC 89627	*Juglans regia*	Shannxi, China	KR045648	KR045727	KU710979	KU710935	KR045689
C. chiangmaiensis	MFLUCC 21-0049	NA	Thailand	NA	NA	MZ451165	MZ451161	MZ451169
C. chrysosperma	CFCC 54081	*Populus simonii*	Gansu, China	MZ702631	NA	NA	OK303613	OK303680
	CFCC 89982	*Ulmus pumila*	Xizang, China	KP281261	KP310805	NA	KP310848	KP310818
C. cinereostroma	CBS:117081	*Eucalyptus globulus*	Chile	KY051841	MH874566	NA	NA	KX964928
	CPC 20832	*Abies alba*	Spain	KY051964	NA	KX965567	KX965206	KX965012
C. cinnamomea	CFCC 53178[T]	*Prunus armeniaca*	Xinjiang, China	MK673054	MK673084	NA	NA	MK672970
C. coryli	CFCC 53162[T]	*Corylus mandshurica*	Beijing, China	MN854450	MN854661	MN850751	MN850758	MN861120
C. corylina	CFCC 54684[T]	*Corylus heterophylla*	Beijing, China	MW839861	NA	MW815951	MW815886	MW883969
	CFCC 54687	*Corylus heterophylla*	Beijing, China	MW839864	NA	MW815954	MW815889	MW883972
C. cotini	MFLUCC 14-1050[T]	*Cotinus coggygria*	Russia	KX430142	KX430143	KX430144	NA	NA

C. cotoneastricola	CF 20197028	*Cotoneaster*sp.	Xizang, China	MK673073	MK673103	MK673013	MK672959	MK672989
	CF 20197031T	*Cotoneaster*sp.	Xizang, China	MK673075	MK673105	MK673015	MK672961	MK672991
C. curreyi	CBS148.42	*Larix sp.*	Switzerland	MH856105	MH867602	NA	NA	NA
C. curvata	MFLUCC 15-0865T	*Salix alba*	Russia	KY417728	KY417762	KY417796	NA	NA
C. curvispora	CFCC 54000T	*Corylus heterophylla*	Beijing, China	MW839851	NA	MW815945	MW815880	MW883963
	CFCC 54679	*Corylus heterophylla*	Beijing, China	MW839856	NA	MW815950	MW815885	MW883968
C. cypri	CBS 118555	*Olea europaea var. africana*	South Africa	DQ243790	NA	NA	NA	KM034893
C. davidiana	CXY 1350T	*Populus davidiana*	Nei Monggol, China	KM034870	NA	NA	NA	KM034902
C. diatrypelloidea	CBS 116826	*Eucalyptus globulus*	Australia	KY051800	KX965264	KX965475	KX965071	KX964906
C. diatrypoides	JacLeuco	*Alnus tenuifolia*	America	JX438611	NA	NA	JX438583	NA
C. diopuiensis	CFCC56804	*Ziziphus jujuba*	China	OR029619	NA	NA	OQ862393	OQ862484
	CFCC56756	*Viburnum opulus subsp. calvescens*	China	OR029618	NA	NA	OQ862392	OQ862483
C. disciformis	CBS 116827	*Eucalyptus grandis*	Uruguay	KY051801	KX965265	KX965476	KX965072	KX964907
	CBS 116828	*Eucalyptus globulus*	Australia	KY051802	KX965266	NA	NA	KX964908
C. discotoma	CFCC 53137 T	*Platycladus orientalis*	Beijing, China	MW418404	MW418416	MW422907	MW422919	MW422931
	CFCC 54368	*Platycladus orientalis*	Beijing, China	MW418405	MW418417	MW422908	MW422920	MW422932
C. donetzica	MFLUCC 15-0864	*Crataegus monogyna*	Russia	KY417729	KY417763	KY417797	NA	NA
	MFLUCC 16-0574T	*Crataegus monogyna*	Russia	KY417731	KY417765	KY417799	NA	NA
C. donglingensis	CFCC 53159 T	*Platycladus orientalis*	Beijing, China	MW418412	MW418424	MW422915	MW422927	MW422939
	CFCC 54371	*Platycladus orientalis*	Beijing, China	MW418413	MW418425	MW422916	MW422928	MW422940
C. elaeagni	CFCC 89632	*Elaeagnus angustifolia*	Ningxia, China	KR045626	KR045706	KU710955	KU710918	KR045667
	CFCC 89633	*Elaeagnus angustifolia*	Ningxia, China	KF765677	KF765693	KU710956	KU710919	KR045668
C. elaeagnicola	CFCC 52882T	*Elaeagnus angustifolia*	China	MK732341	MK732338	MK732347	NA	NA
	CFCC 52883	*Elaeagnus angustifolia*	China	MK732342	MK732339	MK732348	NA	NA
C. eriobotryae	CBS 116846	*Eriobotrya japonica*	India		KX965282	KX965484	KX965084	KX964915
C. erumpens	CFCC 50022	*Prunus padus*	Shanxi, China	MH933627	MH933661	NA	MH933502	MH933569

	MFLUCC 16-0580T	*Salix × fragilis*	Russia	KY417733	KY417767	KY417801	NA	NA
	CFCC 53163	*Prunus padus*	Xinjiang, China	MK673059	MK673089	MK673000	MK672948	MK672975
C. eucalyptina	CBS 116853	*Eucalyptus grandis*	Columbia	KY051822	KX965288	NA	KX965089	KX964918
C. eugeniae	CBS 116835	*Eugenia*	Sulawesi	KY051809	KX965273	NA	KX965079	NA
	CBS 116836	*Eugenia*	Sulawesi	KY051810	KX965274	NA	KX965080	KX964911
C. euonymicola	CFCC 50499T	*Euonymus kiautschovicus*	Shannxi, China	MH933628	MH933662	MH933598	MH933503	MH933570
	CFCC 50500	*Euonymus kiautschovicus*	Shannxi, China	MH933629	MH933663	MH933599	MH933504	MH933571
C. euonymina	CFCC 89993T	*Euonymus kiautschovicus*	Shanxi, China	MH933630	MH933664	MH933600	MH933505	MH933590
	CFCC 89999	*Euonymus kiautschovicus*	Shanxi, China	MH933631	MH933665	MH933601	MH933506	MH933591
C. eutypelloides	CBS 115107	*Man*	Turkey	KY051781	KX965244	KX965465	NA	KX964891
	CPC 19921	*Vitis vinifera*	Iran	KY051956	NA	KX965560	KX965198	KX965004
C. fraxinigena	MFLUCC 14-0868T	*Fraxinus ornus*	Italy	MF190133	MF190078	NA	NA	NA
C. friesii	CBS 194.42	*Abies alba*	Switzerland	KY051899	KX965360	KX965530	KX965150	KX964960
C. fugax	CXY 1371	*Populus simonii*	Jilin, China	KM034852	NA	NA	NA	KM034891
	CXY 1381	*Populus ussuriensis*	Heilongjiang, China	KM034853	NA	NA	NA	KM034890
C. fusispora	NFCCI 4372	NA	India	MN227694	MN250032	NA	NA	NA
C. galegicola	MFLUCC 18-1199T	*Galega officinalis*	Italy	MK912128	MK571756	MN685820	NA	NA
C. gelida	MFLUCC 16-0634	*Cotinus coggygria Scop.*	Russia	KY563245	KY563247	KY563243	NA	NA
C. germanica	CBS 195.42	*Abies alba*	Switzerland	KY051902	KX965361	KX965531	KX965151	KX964961
	CBS 196.42	*Abies alba*	Switzerland	KY051904	KX965363	KX965533	NA	KX964963
C. gigalocus	CFCC 89620T	*Juglans regia*	Qinghai, China	KR045628	KR045708	KU710957	KU710920	KR045669
	CFCC 89621	*Juglans regia*	Qinghai, China	KR045629	KR045709	KU710958	KU710921	KR045670
C. gigaspora	CFCC 50014	*Juniperus procumbens*	Shanxi, China	KR045630	KR045710	KU710959	KU710922	KR045671
	CFCC 89634T	*Salix psammophila*	Shannxi, China	KF765671	KF765687	KU710960	KU710923	KR045672
C. globosa	MFLU 16-2054T	*Abies alba*	Italy	MT177935	MT177962	MT432212	MT454016	NA
C. haidianensis	CFCC 54056	*Euonymus alatus*	Beijing, China	MT360041	NA	MT363987	MT363997	MT364007
	CFCC 54057T	*Euonymus alatus*	Beijing, China	MT360042	NA	MT363988	MT363998	MT364008

C. heveae	MFLUCC 17-0358	*Hevea brasiliensis*	Thailand	OL780505	OL782085	NA	OL944428	NA
C. hippophaës	CFCC 89639	*Hippophaë rhamnoides*	Gansu, China	KR045632	KR045712	KU710961	KU710924	KR045673
	CFCC 89640	*Hippophaë rhamnoides*	Gansu, China	KF765682	KF765698	KU710962	KP310865	KR045674
C. japonica	CFCC 89956	*Prunus cerasifera*	Ningxia, China	KR045624	KR045704	KU710953	KU710916	KR045665
	CFCC 89960	*Prunus cerasifera*	Ningxia, China	KR045625	KR045705	KU710954	KU710917	KR045666
C. junipericola	BBH 42444	*Juniperus communis*	Italy	MF190125	MF190071	NA	MF377579	NA
	MFLU 17-0882[T]	*Juniperus communis*	Italy	MF190126	MF190072	NA	MF377580	NA
C. juniperina	CFCC 50501[T]	*Juniperus przewalskii*	Sichuan, China	MH933632	MH933666	MH933602	MH933507	NA
	CFCC 50502	*Juniperus przewalskii*	Sichuan, China	MH933633	MH933667	MH933603	MH933508	MH933572
C. kantschavelii	CXY 1383	*Populus maximowiczii*	Jilin, China	KM034867	NA	NA	NA	KM034889
	CXY 1386	*Populus maximowiczii*	Chongqing, China	KM034866	NA	NA	NA	KM034888
C. kuanchengensis	CFCC 52464[T]	*Castanea mollissima*	China	MK432616	MK429886	MK578076	NA	NA
	CFCC 52465	*Castanea mollissima*	China	MK432617	MK429887	MK578077	NA	NA
C. kunzei	CBS 114651	*Picea pungens*	Illinois, US	KY051780	KX965243	NA	KX965055	KX964890
	CBS 118093	*Picea pungens*	Michigan, US	KY051855	KX965322	NA	KX965116	KX964941
C. leucosperma	CFCC 89622	*Pyrus bretschneideri*	Gansu, China	KR045616	KR045698	KU710944	KU710911	KR045657
	CFCC 89894	*Pyrus bretschneideri*	Qinghai, China	KR045617	KR045699	KU710945	KU710912	KR045658
C. leucostoma	CFCC 50023	*Cornus alba*	Shanxi, China	KR045635	KR045715	KU710964	KU710926	KR045676
	CFCC 50024	*Prunus pseudocerasus*	Qinghai, China	MH933640	MH933674	MH933605	NA	MH933576
	CFCC 53141	*Prunus sibirica*	Beijing, China	MN854446	MN854657	MN850747	MN850754	MN861116
	CFCC 53156	*Juglans mandshurica*	Beijing, China	MN854447	MN854658	MN850748	MN850755	MN861117
	CFCC 53167	*Prunus armeniaca*	Xinjiang, China	MK673056	MK673086	MK672998	MK672946	MK672972
	CFCC 53169	*Prunus persica*	Beijing, China	MK673080	MK673110	MK673020	MK672966	MK672996
	CFCC 53170	*Prunus persica*	Beijing, China	MK673081	MK673111	MK673021	MK672967	MK672997
	CFCC 54680	*Corylus heterophylla*	Beijing, China	MW839857	NA	MW815955	MW815890	MW883973
	CFCC 54681	*Corylus heterophylla*	Beijing, China	MW839858	NA	MW815956	MW815891	MW883974
	CFCC 54682	*Corylus heterophylla*	Beijing, China	MW839859	NA	MW815957	MW815892	MW883975

	CFCC 54683	*Corylus heterophylla*	Beijing, China	MW839860	NA	MW815958	MW815893	MW883976
C. longistiolata	MFLUCC 16-0628	*Salix × fragilis*	Russia	KY417734	KY417768	KY417802	NA	NA
C. lumnitzericola	MFLUCC 17-0508[T]	*Lumnitzera racernosa*	Thailand	MG975778	MH253453	MH253453	NA	NA
C. mali	CFCC 50028	*Malus pumila*	Gansu, China	MH933641	MH933675	MH933606	MH933513	MH933577
	CFCC 50030	*Malus pumila*	Shannxi, China	MH933643	MH933677	MH933608	MH933524	MH933579
C. mali-spectabilis	CFCC 53181[T]	*Malus spectabilis* 'Royalty'	Xinjiang, China	MK673066	MK673096	MK673006	MK672953	MK672982
C. mali-sylvestris	MFLUCC 16-0638	NA	NA	KY885017	KY885018	KY885020	NA	NA
C. massariana	CBS 141473	*Abies alba*	Switzerland	KY051888	KY051765	NA	KY051759	KY051753
C. melnikii	CFCC 89984	*Rhus typhina*	Xinjiang, China	MH933644	MH933678	MH933609	MH933515	MH933580
	MFLUCC 15-0851	*Malus domestica*	Russia	KY417735	KY417769	KY417803	NA	NA
C. mougeotii	CBS:198.50	*Picea abies*	Norway	KY051910	KX965368	KX965538	KX965158	NA
C. myrtagena	CFCC 52454	*Castanea mollissima*	China	MK432614	MK429884	MK578074	NA	NA
	CFCC 52455	*Castanea mollissima*	China	MK432615	MK429885	MK578075	NA	NA
C. nitschkei	CBS 116854	*Eucalyptus globulus*	Ethiopia	KY051823	KX965289	KX965487	KX965090	NA
	CBS 117605	*Eucalyptus saligna*	Ethiopia	KY051843	KX965310	KX965503	KX965108	KX964930
C. nivea	MFLUCC 15-0860	*Salix acutifolia*	Russia	KY417737	KY417771	KY417805	NA	NA
	CFCC 89641	*Elaeagnus angustifolia*	Ningxia, China	KF765683	KF765699	KU710967	KU710929	KR045679
C. notastroma	NE_TFR5	*Populus tremuloides*	America	JX438632	NA	NA	JX438543	NA
	NE_TFR8	*Populus tremuloides*	America	JX438633	NA	NA	JX438542	NA
C. ochracea	CFCC 53164[T]	*Cotoneaster*sp.	Xinjiang, China	MK673060	MK673090	MK673001	MK672949	MK672976
C. olivacea	CFCC 53175	*Prunus dulcis*	Xinjiang, China	MK673062	MK673092	MK673003	NA	MK672978
	CFCC 53176[T]	*Sorbus tianschanica*	Xinjiang, China	MK673068	MK673098	MK673008	MK672955	MK672984
C. palm	CXY 1276	*Cotinus coggygria*	Beijing, China	JN402990	NA	NA	KJ781296	NA
	CXY 1280[T]	*Cotinus coggygria*	Beijing, China	JN411939	NA	NA	KJ781297	NA
C. parakantschavelii	MFLUCC 15-0857[T]	*Populus × sibirica*	Russia	KY417738	KY417772	KY417806	NA	NA
	MFLUCC 16-0575	*Pyruspyraster*	Russia	KY417739	KY417773	KY417807	NA	NA
C. paraplurivora	FDS-623	*Prunus persica*	Canada	OL640181	OL640123	NA	OL631591	NA

C. parasitica	MFLUCC 15-0507^{T}	*Malus domestica*	Russia	KY417740	KY417774	KY417808	NA	NA
	CFCC 53172	*Malus pumila*	Xinjiang, China	MK673069	MK673099	MK673009	MK672956	MK672985
C. paratranslucens	MFLUCC 15-0506^{T}	*Populus alba var. bolleana*	Russia	KY417741	KY417775	KY417809	NA	NA
	MFLUCC 16-0627	*Populus alba*	Russia	KY417742	KY417776	KY417810	NA	NA
C. pavettae	CBS:145562	*Pavetta revoluta*	South Africa	MK876386	MK876427	MK876483	MK876497	MK876503
C. phialidica	MFLUCC 17-2498	*Alnus glutinosa*	Italy	MT177932	MT177959	MT432209	MT454014	NA
C. phitsanulokensis	MFLUCC 21-0046	NA	Thailand	NA	NA	MZ451168	MZ451164	MZ451172
C. piceae	CFCC 52841^{T}	*Picea crassifolia*	Xinjiang, China	MH820398	MH820391	MH820395	MH820402	MH820387
	CFCC 52842	*Picea crassifolia*	Xinjiang, China	MH820399	MH820392	MH820396	MH820403	MH820388
C. pingbianensis	MFLUCC 18-1204^{T}	*Undefined wood*	Yunnan, China	MK912135	MK571763	MN685826	NA	NA
C. pini	CBS 197.42	*Pinus sylvestris*	Switzerland	KY051908	KX965366	KX965536	KX965155	KX964965
	CPC 28408	*Abies alba*	Switzerland	KY051994	NA	KX965591	NA	KX965041
C. platanicola	MFLU 17-0327	*Platanus hybrida*	Italy	MH253451	MH253452	MH253450	NA	NA
C. platycladi	CFCC 50504^{T}	*Platycladus orientalis*	Yunnan, China	MH933645	MH933679	MH933610	MH933516	MH933581
	CFCC 50505	*Platycladus orientalis*	Yunnan, China	MH933646	MH933680	MH933611	MH933517	MH933582
C. platycladicola	CFCC 50038^{T}	*Platycladus orientalis*	Gansu, China	KT222840	MH933682	MH933613	MH933519	MH933584
	CFCC 50039	*Platycladus orientalis*	Gansu, China	KR045642	KR045721	KU710973	KU710931	KR045683
C. populi	CFCC 55471	*Populus* sp.	Gansu, China	MZ702608	NA	OK303529	OK303590	OK303657
	CFCC 55472^{T}	*Populus* sp.	Gansu, China	MZ702609	NA	OK303530	OK303591	OK303658
C. populina	CFCC 89644^{T}	*Salix psammophila*	Shannxi, China	KF765686	KF765702	KU710969	KU710930	KR045681
C. populinopsis	CFCC 50032^{T}	*Sorbus aucuparia*	Ningxia, China	MH933648	MH933683	MH933614	MH933520	MH933585
	CFCC 50033	*Sorbus aucuparia*	Ningxia, China	MH933649	MH933684	MH933615	MH933521	MH933586
C. predappioensis	MFLUCC 17-2458^{T}	*Platanus hybrida*	Italy	MG873484	MG873480	NA	NA	NA
C. pruinopsis	CFCC 50034^{T}	*Ulmus pumila*	Shannxi, China	KP281259	KP310806	KU710970	KP310849	KP310819
	CFCC 50035	*Ulmus pumila*	Jilin, China	KP281260	KP310807	KU710971	KP310850	KP310820
C. pruinosa	CFCC 50036	*Syringa oblata*	Qinghai, China	KP310800	KP310802	NA	KP310845	KP310815

	CFCC 50037	*Syringa oblata*	Qinghai, China	MH933650	MH933685	NA	MH933522	MH933589
C. prunicola	MFLU 17-0995[T]	*Prunus* sp.	Italy	MG742350	MG742351	MG742352	MG742354	NA
C. pruni-mume	CFCC 53179	*Prunus armeniaca*	Xinjiang, China	MK673057	MK673087	NA	MK672947	MK672973
	CFCC 53180[T]	*Prunus mume*	Xinjiang, China	MK673067	MK673097	MK673007	MK672954	MK672983
C. pubescentis	MFLUCC 18-1201[T]	*Quercus pubescens*	Italy	MK912130	MK571758	MN685821	NA	NA
C. pustulata	CBS 202.42	*Abies alba*	Switzerland	KY051914	KX965372	NA	NA	NA
C. quercicola	MFLU 17-0881	*Quercus* sp.	Italy	MF190129	MF190074	NA	NA	NA
	MFLUCC 14-0867[T]	*Quercus* sp.	Italy	MF190128	MF190073	NA	NA	NA
C. rhodophila	CBS 349.69	*Fraxinus excelsior*	Netherlands	KY051928	KX965385	KX965546	KX965172	KX964982
C. ribis	CFCC 50026	*Ulmus pumila*	Qinghai, China	KP281267	KP310813	KU710972	KP310856	KP310826
	CFCC 50027	*Ulmus pumila*	Qinghai, China	KP281268	KP310814	NA	KP310857	KP310827
C. rosae	MFLU 17-0885	*Rosa canina*	Italy	MF190131	MF190076	NA	NA	NA
C. rosarum	MFLUCC 16-0625	*Acer platanoides L.*	Russia	KY563246	KY563248	KY563244	NA	NA
C. rosicola	CF 20197024[T]	*Rosa* sp.	Xizang, China	MK673079	MK673109	MK673019	MK672965	MK672995
C. rosigena	MFLUCC 18-0921[T]	*Rosa* sp.	Russia	MN879872	MN879873	NA	NA	NA
C. rostrata	CFCC 89909	*Salix cupularis*	Gansu, China	KR045643	KR045722	KU710974	KU710932	KR045684
	CFCC 89910	*Salix cupularis*	Gansu, China	KR045644	NA	KU710975	KU710933	NA
C. rusanovii	MFLUCC 15-0853	*Populus × sibirica*	Russia	KY417743	KY417777	KY417811	NA	NA
	MFLUCC 15-0854[T]	*Salix babylonica*	Russia	KY417744	KY417778	KY417812	NA	NA
C. sacchari	CBS 160.33	*Saccharum officinarum*	India	KY051891	KX965301	KX965496	KX965100	KX964925
C. salicacearum	MFLUCC 15-0509	*Salix alba*	Russia	KY417746	KY417780	KY417814	NA	NA
	MFLUCC 15-0861	*Salix × fragilis*	Russia	KY417745	KY417779	KY417813	NA	NA
C. salicicola	MFLUCC 14-1052[T]	*Salix alba*	Russia	KU982636	KU982635	NA	NA	NA
	MFLUCC 15-0866	*Salix* sp.	Thailand	KY417749	KY417783	KY417817	NA	NA
C. salicina	MFLUCC 15-0862	*Salix alba*	Russia	KY417750	KY417784	KY417818	NA	NA
	MFLUCC 16-0637	*Salix × fragilis*	Russia	KY417751	KY417785	KY417819	NA	NA
C. salicis	CBS 109754	*Salix fragilis*	Austria	KY051772	KX965237	NA	KX965051	KX964884
C. schulzeri	CFCC 50042	*Malus pumila*	Gansu, China	KR045650	KR045729	KU710981	KU710937	KR045691

C. shoreae	MFLUCC 21-0047	NA	Thailand	NA	NA	MZ451166	MZ451162	MZ451170
	MFLUCC 21-0048	NA	Thailand	NA	NA	MZ451167	MZ451163	MZ451171
C. sibiraeae	CFCC 50045[T]	*Sibiraea angustata*	Gansu, China	KR045651	KR045730	KU710982	KU710938	KR045692
	CFCC 50046	*Sibiraea angustata*	Gansu, China	KR045652	KR045731	KU710983	KU710939	KR045693
C. sophorae	CFCC 50047	*Styphnolobium japonicum*	Shanxi, China	KR045653	KR045732	KU710984	KU710940	KR045694
	CFCC 50048	*Magnolia grandiflora*	Shanxi, China	MH820401	MH820394	MH820397	MH820405	MH820390
C. sophoricola	CFCC 89596	*Styphnolobium japonicum* var. *pendula*	Gansu, China	KR045656	KR045735	KU710987	KU710943	KR045697
	CFCC 89595[T]	*Styphnolobium japonicum* var. *pendula*	Gansu, China	KR045655	KR045734	KU710986	KU710942	KR045696
C. sophoriopsis	CFCC 54070	*Populus simonii*	Inner Mongolia, China	MZ702623	NA	OK303544	OK303605	OK303672
	CFCC 89600	*Styphnolobium japonicum*	Gansu, China	KR045623	KP310804	KU710951	KU710915	KP310817
C. sorbi	MFLUCC 16-0631[T]	*Sorbus aucuparia*	Russia	KY417752	KY417786	KY417820	NA	NA
C. sorbicola	MFLUCC 16-0584[T]	*Acer pseudoplatanus*	Russia	KY417755	KY417789	KY417823	NA	NA
	MFLUCC 16-0633	*Cotoneaster melanocarpus*	Russia	KY417758	KY417792	KY417826	NA	NA
C. sorbina	CF 20197660[T]	*Sorbus tianschanica*	Xinjiang, China	MK673052	MK673082	NA	MK672943	MK672968
C. spiraeae	CFCC 50049[T]	*Spiraea salicifolia*	Gansu, China	MG707859	MG707643	MG708199	NA	NA
	CFCC 50050	*Spiraea salicifolia*	Gansu, China	MG707860	MG707644	MG708200	NA	NA
C. spiraeicola	CFCC 53138[T]	*Spiraea salicifolia*	Beijing, China	MN854448	MN854659	MN850749	MN850756	MN861118
	CFCC 53139	*Tilia nobilis*	Beijing, China	MN854449	MN854660	MN850750	MN850757	MN861119
C. subclypeata	CPC 28406	*Betula*	Switzerland	KY051992	KX965450	NA	NA	KX965039
C. tamaricicola	CFCC 50507	*Rosa multifolora*	Yunnan, China	MH933651	MH933686	MH933616	MH933525	MH933587
	CFCC 50508[T]	*Tamarix chinensis*	Yunnan, China	MH933652	MH933687	MH933617	MH933523	MH933588
C. tanaitica	MFLUCC 14-1057[T]	*Betula pubescens*	Russia	KT459411	KT459412	NA	NA	NA
C. thailandica	MFLUCC 17-0262[T]	*Xylocarpus moluccensis*	Thailand	MG975776	MH253455	MH253463	NA	NA
	MFLUCC 17-0263[T]	*Xylocarpus moluccensis*	Thailand	MG975777	MH253456	MH253464	NA	NA

C. tibetensis	CF 20197026	*Cotoneaster*sp.	Xizang, China	MK673076	MK673106	MK673016	MK672962	MK672992
	CF 20197032T	*Cotoneaster*sp.	Xizang, China	MK673078	MK673108	MK673018	MK672964	MK672994
C. tibouchinae	CPC 26333T	*Tibouchina semidecandra*	France	KX228284	KX228335	NA	NA	NA
C. translucens	CXY 1351	*Populus davidiana*	Inner Mongolia, China	KM034874	NA	NA	NA	KM034895
	CXY 1359	*Populus* × Beijingensis	Beijing, China	KM034871	NA	NA	NA	KM034894
C. tritici	CPC 19924	*Vitis vinifera*	Iran	KY051959	NA	KX965563	KX965201	KX965007
C. ulmi	MFLUCC 15-0863T	*Ulmus minor*	Russia	KY417759	KY417793	KY417827	NA	NA
C. valsoidea	CBS:117003	*Eucalyptus grandis*	Sumatra	KY051832	KX965298	KX965494	KX965097	NA
C. variostromatica	CBS 116858	*Eucalyptus globulus*	Australia	KY051828	KX965293	KX965491	NA	KX964921
C. verrucosa	CFCC 53157 T	*Platycladus orientalis*	Beijing, China	MW418408	MW418420	MW422911	MW422923	MW422935
	CFCC 54369	*Platycladus orientalis*	Beijing, China	MW418409	MW418421	MW422912	MW422924	MW422936
C. vinacea	CBS 141585T	*Vitis interspecific*	America	KX256256	NA	NA	KX256277	KX256235
C. viridistroma	CBS 202.36T	*Cercis canadensis* Castigl.	America	MN172408	MN172388	NA	MN271853	NA
C. viticola	CyT2	*Vitis interspecific*	America	KX256238	NA	NA	KX256259	KX256217
	CBS 141586T	*Vitis vinifera*	America	KX256239	NA	NA	KX256260	KX256218
C. xinglongensis	CFCC 52458	*Castanea mollissima*	China	MK432622	MK429892	MK578082	NA	NA
	CFCC 52459	*Castanea mollissima*	China	MK432623	MK429893	MK578083	NA	NA
C. xinjiangensis	CFCC 53182	*Rosa* sp.	Xinjiang, China	MK673064	MK673094	MK673004	MK672951	MK672980
	CFCC 53183T	*Rosa* sp.	Xinjiang, China	MK673065	MK673095	MK673005	MK672952	MK672981
C. xylocarpi	MFLUCC 17-0251T	*Xylocarpus granatum*	Thailand	MG975775	MH253454	MH253454	NA	NA
Diaporthe eres	CBS:145040	*Lactuca sativa*	Netherlands	MK442579	MK442521	MK442663	MK442693	MK442731
D. vaccinii	CBS 160.32	*Vaccinium macrocarpon*	America	KC343228	MH866710	NA	KC343954	KC344196
