# Supplementary material for: Diversity of Cytospora Species Associated with Trunk Diseases of Prunus persica (Peach) in Northern China
Source: J Fungi (Basel). 2024 Dec 5;10(12):843. doi: 10.3390/jof10120843 (PMC11678414; doi:10.3390/jof10120843)
Supplement: Supplementary file 1 [file jof-10-00843-s001.zip › Supplementary Materials-Table S2.pdf]

Table S2. Isolates and GenBank accession numbers of sequences obtained from this study

Species	Strain	ITS	LSU	*rpb2*	*tef1-α*	*tub2*
C. ailanthicola	JZB3670084	NA	OR244338	NA	OR253592	OR253603
	JZB3670087	OR244335	OR244341	OR253600	OR253595	NA
	JZB3670090	OR244337	OR244344	OR253602	OR253597	NA
C. erumpens	JZB3670060	OR077475	OR226490	OR228347	OR194933	OR228390
	JZB3670064	OR077478	OR226494	OR228349	OR194936	OR228393
	JZB3670066	OR077479	OR226496	OR228351	OR194938	OR228395
C. leucosperma	JZB3670055	OR077471	OR195730	OR228343	OR194982	OR195024
	JZB3670056	OR077472	OR195731	OR228344	OR194983	OR195025
	JZB3670057	OR077473	OR195732	OR228345	OR194984	NA
C. leucostoma	JZB3670014	NA	OR226508	OR228361	OR194950	OR194993
	JZB3670016	OR077491	OR226510	NA	NA	NA
	JZB3670017	OR077492	OR226511	OR228363	NA	NA
	JZB3670036	OR077502	OR226530	OR228377	NA	OR195012
	JZB3670040	NA	OR226534	OR228379	OR194971	OR195013
	JZB3670049	OR077508	OR226543	NA	OR194978	OR195021
	JZB3670071	OR251446	OR256219	NA	OR253607	NA
	JZB3670078	OR251456	OR256229	NA	OR253617	OR253643
	JZB3670079	OR251457	OR256230	OR253631	OR253618	OR253644
	JZB3670098	PQ059100	PQ059125	PQ061555	NA	PQ061574
	JZB3670099	PQ059101	PQ059126	PQ061556	PQ061547	PQ061575
	JZB3670100	PQ059102	PQ059127	PQ061557	NA	PQ061576
	JZB3670133	PQ059105	PQ059129	PQ061559	PQ061548	PQ061579
	JZB3670140	PQ059112	PQ059134	NA	NA	NA
	JZB3670146	PQ059117	PQ059140	PQ061569	PQ061553	PQ061590
C. qinanensis	JZB3670103	PQ013667	NA	NA	NA	PQ053500
	JZB3670104	NA	NA	NA	NA	PQ053501
	JZB3670105	PQ013668	NA	NA	NA	PQ053502
	JZB3670106	NA	NA	NA	NA	PQ053503
	JZB3670107	PQ013669	PQ013712	PQ053484	NA	PQ053504
	JZB3670108	PQ013670	PQ013713	PQ053485	NA	PQ053505
	JZB3670110	NA	NA	NA	NA	PQ053506
	JZB3670111	PQ013671	NA	NA	NA	PQ053507
C. gansuensis	JZB3670130	PQ013683	PQ032288	PQ053497	PQ044439	PQ053519
	JZB3670131	PQ013684	PQ032289	PQ053498	PQ044440	PQ053520
	JZB3670132	PQ013685	PQ032290	PQ053499	NA	PQ053521
