# Supplementary material for: Diversity of Cytospora Species Associated with Trunk Diseases of Prunus persica (Peach) in Northern China
Source: J Fungi (Basel). 2024 Dec 5;10(12):843. doi: 10.3390/jof10120843 (PMC11678414; doi:10.3390/jof10120843)
Supplement: Supplementary file 1 [file jof-10-00843-s001.zip › Supplementary Materials-Table S3.pdf]

Table S3. Isolates obtained in this study

Organism	Isolate	Isolation-Source	Geo loc name	Host
C. leucostoma	JZB3670006	branch	Pinggu District, Beijing City, China	*Prunus persica*
C. leucostoma	JZB3670007	branch	Pinggu District, Beijing City, China	*Prunus persica*
C. leucostoma	JZB3670008	branch	Pinggu District, Beijing City, China	*Prunus persica*
C. leucostoma	JZB3670009	branch	Pinggu District, Beijing City, China	*Prunus persica*
C. leucostoma	JZB3670010	branch	Pinggu District, Beijing City, China	*Prunus persica*
C. leucostoma	JZB3670011	branch	Pinggu District, Beijing City, China	*Prunus persica*
C. leucostoma	JZB3670012	branch	Pinggu District, Beijing City, China	*Prunus persica*
C. leucostoma	JZB3670013	branch	Pinggu District, Beijing City, China	*Prunus persica*
C. leucostoma	JZB3670014	branch	Pinggu District, Beijing City, China	*Prunus persica*
C. leucostoma	JZB3670015	branch	Pinggu District, Beijing City, China	*Prunus persica*
C. leucostoma	JZB3670016	branch	Pinggu District, Beijing City, China	*Prunus persica*
C. leucostoma	JZB3670017	branch	Pinggu District, Beijing City, China	*Prunus persica*
C. leucostoma	JZB3670018	branch	Gansu, China	*Prunus persica*
C. leucostoma	JZB3670019	branch	Gansu, China	*Prunus persica*
C. leucostoma	JZB3670020	branch	Gansu, China	*Prunus persica*
C. leucostoma	JZB3670021	branch	Gansu, China	*Prunus persica*
C. leucostoma	JZB3670022	branch	Liaoning, China	*Prunus persica*
C. leucostoma	JZB3670023	branch	Liaoning, China	*Prunus persica*
C. leucostoma	JZB3670024	branch	Liaoning, China	*Prunus persica*
C. leucostoma	JZB3670025	branch	Liaoning, China	*Prunus persica*
C. leucostoma	JZB3670026	branch	Liaoning, China	*Prunus persica*
C. leucostoma	JZB3670027	branch	Liaoning, China	*Prunus persica*
C. leucostoma	JZB3670028	branch	Liaoning, China	*Prunus persica*
C. leucostoma	JZB3670029	branch	Liaoning, China	*Prunus persica*
C. leucostoma	JZB3670030	branch	Liaoning, China	*Prunus persica*
C. leucostoma	JZB3670031	branch	Liaoning, China	*Prunus persica*
C. leucostoma	JZB3670032	branch	Liaoning, China	*Prunus persica*
C. leucostoma	JZB3670033	branch	Liaoning, China	*Prunus persica*
C. leucostoma	JZB3670034	branch	Shunyi District, Beijing City,	*Prunus persica*

			China	
C. leucostoma	JZB3670035	branch	Shunyi District, Beijing City, China	*Prunus persica*
C. leucostoma	JZB3670036	branch	Shunyi District, Beijing City, China	*Prunus persica*
C. leucostoma	JZB3670037	branch	Shunyi District, Beijing City, China	*Prunus persica*
C. leucostoma	JZB3670038	branch	Shunyi District, Beijing City, China	*Prunus persica*
C. leucostoma	JZB3670039	branch	Shunyi District, Beijing City, China	*Prunus persica*
C. leucostoma	JZB3670040	branch	Shunyi District, Beijing City, China	*Prunus persica*
C. leucostoma	JZB3670041	branch	Shunyi District, Beijing City, China	*Prunus persica*
C. leucostoma	JZB3670042	branch	Shunyi District, Beijing City, China	*Prunus persica*
C. leucostoma	JZB3670043	branch	Shunyi District, Beijing City, China	*Prunus persica*
C. leucostoma	JZB3670044	branch	Shunyi District, Beijing City, China	*Prunus persica*
C. leucostoma	JZB3670045	branch	Shunyi District, Beijing City, China	*Prunus persica*
C. leucostoma	JZB3670046	branch	Shunyi District, Beijing City, China	*Prunus persica*
C. leucostoma	JZB3670047	branch	Shunyi District, Beijing City, China	*Prunus persica*
C. leucostoma	JZB3670048	branch	Shunyi District, Beijing City, China	*Prunus persica*
C. leucostoma	JZB3670049	branch	Shunyi District, Beijing City, China	*Prunus persica*
C. leucostoma	JZB3670050	branch	Shunyi District, Beijing City, China	*Prunus persica*
C. leucostoma	JZB3670051	branch	Shunyi District, Beijing City, China	*Prunus persica*
C. leucostoma	JZB3670052	branch	Shunyi District, Beijing City, China	*Prunus persica*
C. leucostoma	JZB3670053	branch	Shunyi District, Beijing City, China	*Prunus persica*
C. leucostoma	JZB3670054	branch	Shunyi District, Beijing City, China	*Prunus persica*
C. leucosperma	JZB3670055	branch	Shunyi District, Beijing City, China	*Prunus persica*
C. leucosperma	JZB3670056	branch	Shunyi District, Beijing City,	*Prunus persica*

			China	
C. leucosperma	JZB3670057	branch	Shunyi District, Beijing City, China	*Prunus persica*
C. erumpens	JZB3670058	branch	Jilin, China	*Prunus persica*
C. erumpens	JZB3670059	branch	Jilin, China	*Prunus persica*
C. erumpens	JZB3670060	branch	Jilin, China	*Prunus persica*
C. erumpens	JZB3670061	branch	Jilin, China	*Prunus persica*
C. erumpens	JZB3670062	branch	Jilin, China	*Prunus persica*
C. erumpens	JZB3670063	branch	Jilin, China	*Prunus persica*
C. erumpens	JZB3670064	branch	Jilin, China	*Prunus persica*
C. erumpens	JZB3670065	branch	Jilin, China	*Prunus persica*
C. erumpens	JZB3670066	branch	Jilin, China	*Prunus persica*
C. erumpens	JZB3670067	branch	Jilin, China	*Prunus persica*
C. erumpens	JZB3670068	branch	Jilin, China	*Prunus persica*
C. erumpens	JZB3670069	branch	Jilin, China	*Prunus persica*
C. leucostoma	JZB3670070	branch	Liaoning, China	*Prunus persica*
C. leucostoma	JZB3670071	branch	Liaoning, China	*Prunus persica*
C. leucostoma	JZB3670072	branch	Liaoning, China	*Prunus persica*
C. leucostoma	JZB3670073	branch	Liaoning, China	*Prunus persica*
C. leucostoma	JZB3670074	branch	Liaoning, China	*Prunus persica*
C. leucostoma	JZB3670075	branch	Liaoning, China	*Prunus persica*
C. leucostoma	JZB3670076	branch	Liaoning, China	*Prunus persica*
C. leucostoma	JZB3670077	branch	Liaoning, China	*Prunus persica*
C. leucostoma	JZB3670078	branch	Liaoning, China	*Prunus persica*
C. leucostoma	JZB3670079	branch	Liaoning, China	*Prunus persica*
C. leucostoma	JZB3670080	branch	Pinggu District, Beijing City, China	*Prunus persica*
C. leucostoma	JZB3670081	branch	Pinggu District, Beijing City, China	*Prunus persica*
C. leucostoma	JZB3670082	branch	Pinggu District, Beijing City, China	*Prunus persica*
C. leucostoma	JZB3670083	branch	Pinggu District, Beijing City, China	*Prunus persica*
C. ailanthicola	JZB3670084	branch	Pinggu District, Beijing City, China	*Prunus persica*
C. ailanthicola	JZB3670085	branch	Pinggu District, Beijing City, China	*Prunus persica*
C. ailanthicola	JZB3670086	branch	Pinggu District, Beijing City, China	*Prunus persica*
C. ailanthicola	JZB3670087	branch	Pinggu District, Beijing City, China	*Prunus persica*
C. ailanthicola	JZB3670088	branch	Pinggu District, Beijing City, China	*Prunus persica*
C. ailanthicola	JZB3670089	branch	Pinggu District, Beijing City,	*Prunus persica*

			China	
C. ailanthicola	JZB3670090	branch	Pinggu District, Beijing City, China	*Prunus persica*
C. leucostoma	JZB3670091	branch	Liaoning, China	*Prunus persica*
C. leucostoma	JZB3670092	branch	Liaoning, China	*Prunus persica*
C. leucostoma	JZB3670093	branch	Liaoning, China	*Prunus persica*
C. leucostoma	JZB3670094	branch	Gansu, China	*Prunus persica*
C. leucostoma	JZB3670095	branch	Gansu, China	*Prunus persica*
C. leucostoma	JZB3670096	branch	Gansu, China	*Prunus persica*
C. leucostoma	JZB3670097	branch	Gansu, China	*Prunus persica*
C. leucostoma	JZB3670098	branch	Gansu, China	*Prunus persica*
C. leucostoma	JZB3670099	branch	Gansu, China	*Prunus persica*
C. leucostoma	JZB3670100	branch	Gansu, China	*Prunus persica*
C. leucostoma	JZB3670101	branch	Gansu, China	*Prunus persica*
C. leucostoma	JZB3670102	branch	Gansu, China	*Prunus persica*
C. qinanensis	JZB3670103	branch	Gansu, China	*Prunus persica*
C. qinanensis	JZB3670104	branch	Gansu, China	*Prunus persica*
C. qinanensis	JZB3670105	branch	Gansu, China	*Prunus persica*
C. qinanensis	JZB3670106	branch	Gansu, China	*Prunus persica*
C. qinanensis	JZB3670107	branch	Gansu, China	*Prunus persica*
C. qinanensis	JZB3670108	branch	Gansu, China	*Prunus persica*
C. qinanensis	JZB3670109	branch	Gansu, China	*Prunus persica*
C. qinanensis	JZB3670110	branch	Gansu, China	*Prunus persica*
C. qinanensis	JZB3670111	branch	Gansu, China	*Prunus persica*
C. gansuensis	JZB3670130	branch	Gansu, China	*Prunus persica*
C. gansuensis	JZB3670131	branch	Gansu, China	*Prunus persica*
C. gansuensis	JZB3670132	branch	Gansu, China	*Prunus persica*
C. leucostoma	JZB3670133	branch	Shandong, China	*Prunus persica*
C. leucostoma	JZB3670134	branch	Shandong, China	*Prunus persica*
C. leucostoma	JZB3670135	branch	Shandong, China	*Prunus persica*
C. leucostoma	JZB3670136	branch	Shandong, China	*Prunus persica*
C. leucostoma	JZB3670137	branch	Shandong, China	*Prunus persica*
C. leucostoma	JZB3670138	branch	Shandong, China	*Prunus persica*
C. leucostoma	JZB3670139	branch	Shandong, China	*Prunus persica*
C. leucostoma	JZB3670140	branch	Shandong, China	*Prunus persica*
C. leucostoma	JZB3670141	branch	Shandong, China	*Prunus persica*
C. leucostoma	JZB3670142	branch	Shandong, China	*Prunus persica*
C. leucostoma	JZB3670143	branch	Shandong, China	*Prunus persica*
C. leucostoma	JZB3670144	branch	Shandong, China	*Prunus persica*
C. leucostoma	JZB3670145	branch	Shandong, China	*Prunus persica*
C. leucostoma	JZB3670146	branch	Shandong, China	*Prunus persica*
C. leucostoma	JZB3670147	branch	Shandong, China	*Prunus persica*
C. leucostoma	JZB3670148	branch	Shandong, China	*Prunus persica*
C. leucostoma	JZB3670149	branch	Shandong, China	*Prunus persica*

C. leucostoma	JZB3670150	branch	Shandong, China	*Prunus persica*
